# Supplementary material for: Cytotoxic CD8+ T cells target citrullinated antigens in rheumatoid arthritis
Source: Nat Commun. 2023 Jan 19;14:319. doi: 10.1038/s41467-022-35264-8 (PMC9852471; doi:10.1038/s41467-022-35264-8)
Supplement: Supplementary file 1 — Supplementary Information [file 41467_2022_35264_MOESM1_ESM.pdf]

Supplementary information

Cytotoxic $CD8^+$ T cells target citrullinated antigens in rheumatoid arthritis

Authors: Jae-Seung Moon[#1,2], Shady Younis[#1,2,3], Nitya S. Ramadoss[1,2], Radhika Iyer[1,2], Khushboo Sheth[1,2], Orr Sharpe[1,2], Navin L. Rao[4], Stephane Becart[5], Julie A. Carman[4], Eddie A. James[6], Jane H. Buckner[6], Kevin D. Deane[7], V. Michael Holers[7], Susan M. Goodman[8,9], Laura T. Donlin[8,9], Mark M. Davis[3,10], William H. Robinson*[1,2,3]

Affiliations:

[1]Division of Immunology and Rheumatology, Stanford University, Stanford, CA 94305, USA

[2]VA Palo Alto Health Care System, Palo Alto, CA, 94304, USA

[3]Institute for Immunity, Transplantation and Infection, Stanford University, Stanford, CA, USA

[4]Immunology Discovery, Janssen Research and Development LLC, Spring House, PA 19477, USA

[5]Immunology Discovery, Janssen Research and Development LLC, San Diego, CA 92121, USA

[6]Benaroya Research Institute, Seattle, WA 98101, USA

[7]Division of Rheumatology, University of Colorado School of Medicine, Aurora, CO 80220, USA

[8]Hospital for Special Surgery, New York, NY 10021, USA

[9]Weill Cornell Medicine, New York, NY 10021, USA

[10]Department of Microbiology and Immunology, Stanford University, Stanford, CA 94305, USA

[#]These authors contributed equally: Jae-Seung Moon, Shady Younis

*To whom correspondence should be addressed: w.robinson@stanford.edu

Supplementary Figures

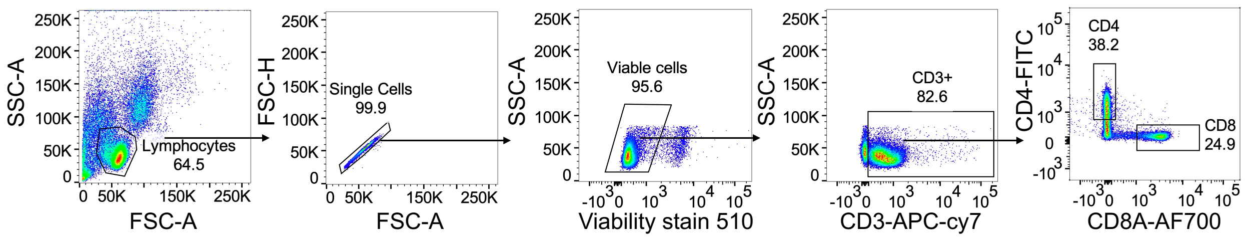

Supplementary Fig. 1: Flow cytometric analysis of $CD8^+$ T cells in ACPA+ RA patient blood. Representative data from ACPA+ RA PBMCs are displayed as flow cytometric plots, and the gating parameters for the $CD8^+$ T cell population indicated.

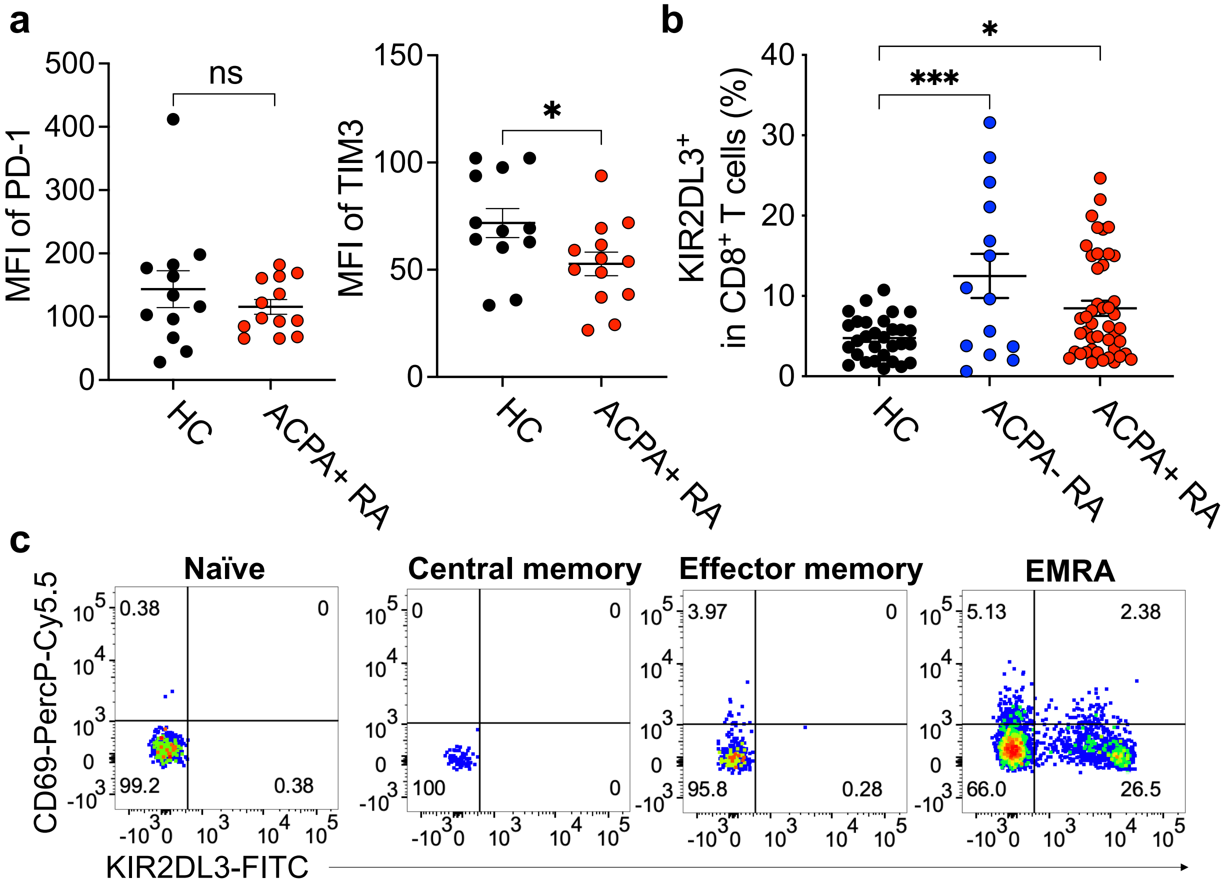

Supplementary Fig. 2: Phenotypic characterization of $CD8^+$ T cells in ACPA+ RA patient blood. a Expression of PD-1 or TIM3 in $CD8^+$ T cells from PBMCs from healthy controls (*n* = 12) or ACPA+ RA patients (*n* = 13) measured by flow cytometry. $^{*}P$=0.0376. **b** Percentage of $KIR2DL3^+CD8^+$ T cells in HC (*n* = 30), ACPA- RA (*n* = 14), or ACPA+ RA (*n* = 45) analyzed by flow cytometry ($^{***}P$=0.0007, or $^{*}P$=0.0355). **c** Representative dot plots showing expression levels of CD69 and KIR2DL3 in $CD8^+$ T memory subsets (naïve, CM, EM, or EMRA). Data are presented as means ± SEM. $^{*}P < 0.05$ or $^{***}P < 0.001$ by two-tailed unpaired *t*-test (**a**) or one-way ANOVA (**b**) with Tukey's multiple comparisons test. ns, not significant. Source data are provided as a Source Data file. HC healthy control, ACPA anti-citrullinated protein antibodies, RA rheumatoid arthritis, MFI median fluorescence intensity.

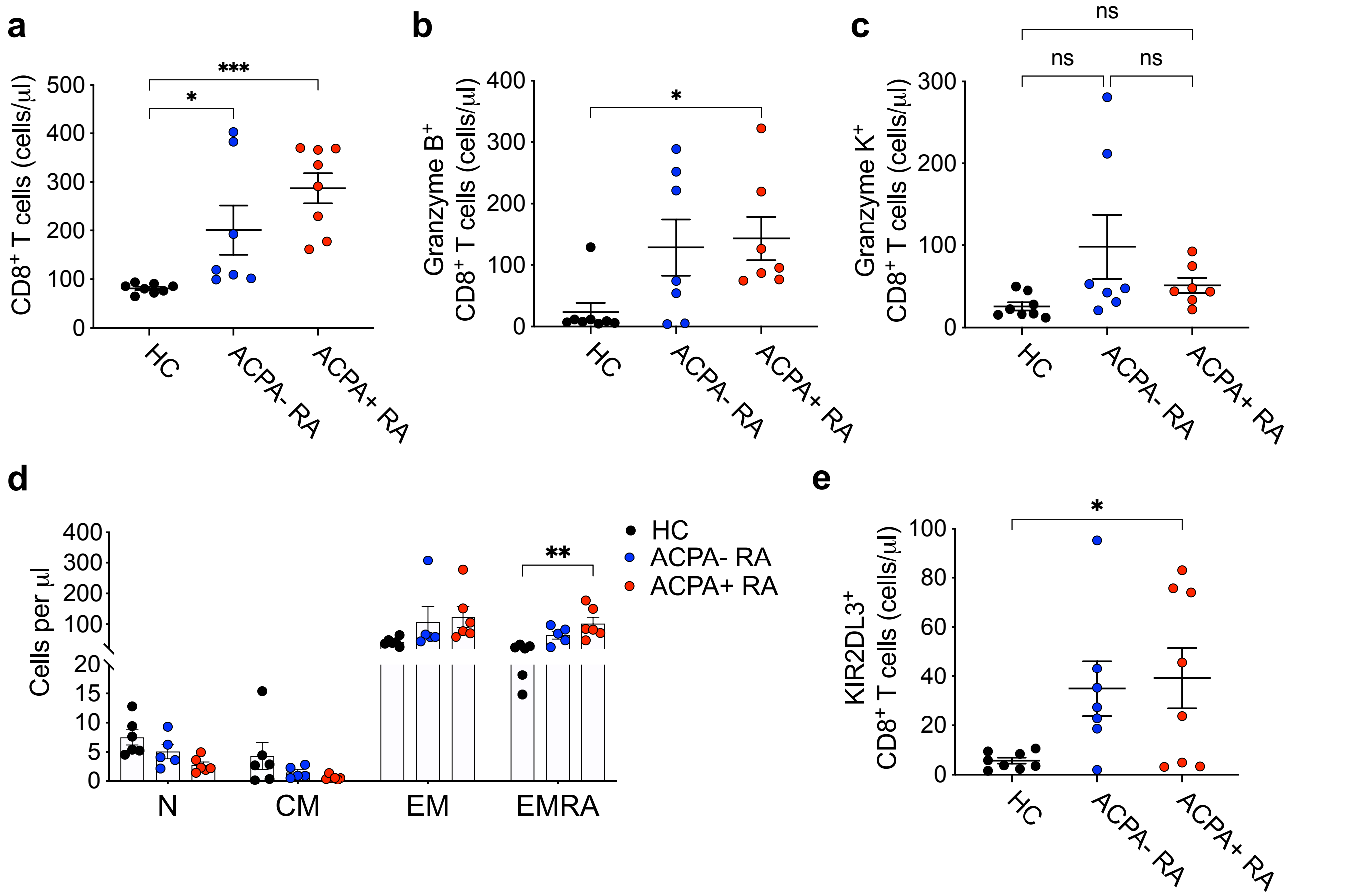

Supplementary Fig. 3: Absolute cell counts of $CD8^+$ T cells in ACPA+ RA patients. Absolute cell numbers of total $CD8^+$ T cells (**a,** HC n = 8, ACPA- RA n = 7, ACPA+ RA n = 8, $^*P$=0.0466, or $^{***}P$=0.0005), granzyme B-expressing $CD8^+$ T cells (**b,** HC n = 8, ACPA- RA n = 7, ACPA+ RA n = 7, $^*P$=0.0473), or granzyme K (**c,** HC n = 8, ACPA- RA n = 7, ACPA+ RA n = 7)-expressing $CD8^+$ T cells, memory $CD8^+$ T cells (**d,** HC n = 6, ACPA- RA n = 5, ACPA+ RA n = 5, $^*P$=0.006), or $KIR2DL3^+CD8^+$ T cells (**e,** HC n = 8, ACPA- RA n = 7, ACPA+ RA n = 8, $^*P$=0.0484) per 1 µl assessed by counting beads in each PBMC. Data are presented as means ± SEM. $^*P < 0.05$, $^{**}P < 0.01$, or $^{***}P < 0.001$ by ordinary one-way ANOVA (**a-c, e**) or two-way ANOVA (**d**) with Tukey's multiple comparisons test. ns, not significant. Source data are provided as a Source Data file. HC healthy control, ACPA anti-citrullinated protein antibodies, RA rheumatoid arthritis.

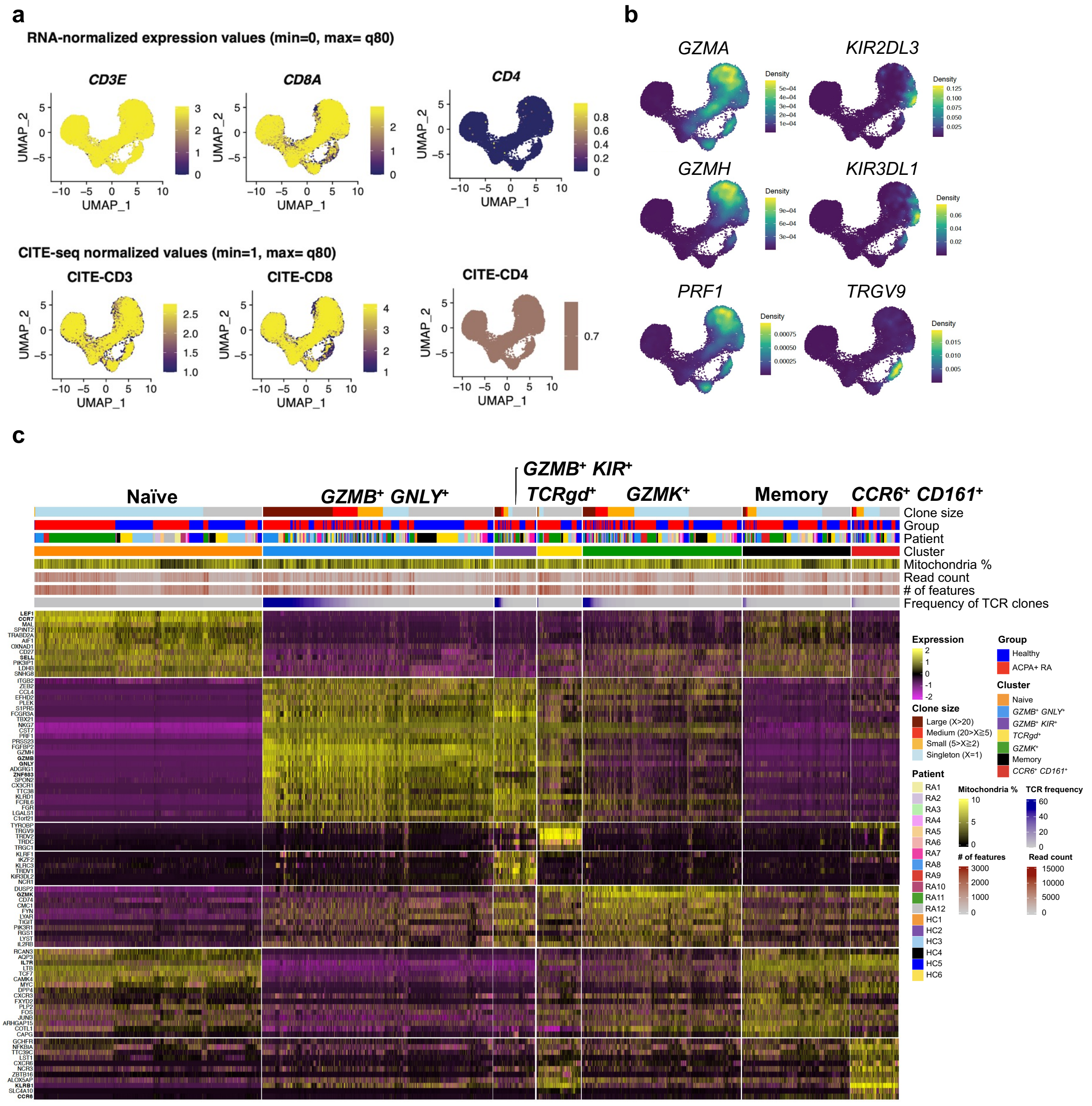

Supplementary Fig. 4: Expression of cell type marker genes in CD8^{+} T cells from ACPA+ RA and healthy control blood. **a** mRNA and CITE-seq expression of marker genes (*CD3E*, *CD8A*, and *CD4*) used to identify and confirm analysis of CD8^{+} T cells. Color indicates the expression level of each marker gene. **b** Density plots display marker genes including *GZMA*, *KIR2DL3* or *TRGV9*) in CD8^{+} T cells. **c** Heatmap of select differentially expressed gene signatures (log2 Fold Change > 0.8) in each cluster with bars; clonal size, patients, clusters, mitochondrial gene percentage, read count, number of features in each cell and frequency of TCR clone. ACPA anti-citrullinated protein antibodies, RA rheumatoid arthritis.

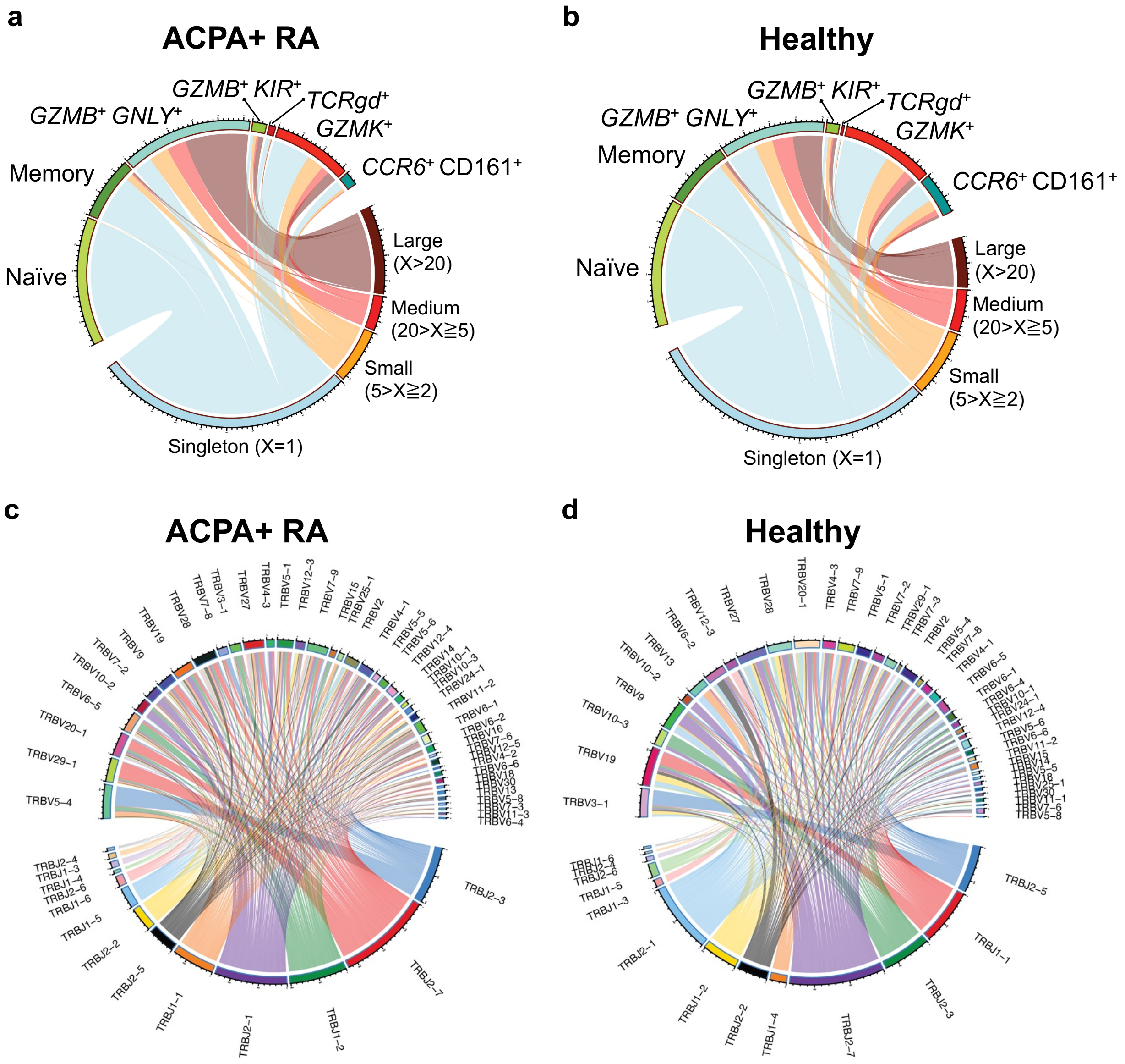

Supplementary Fig. 5: CD8$^+$ T cells expressing cytotoxic mediators are clonally expanded in ACPA+ RA blood. a, b Chord diagrams representing the contribution of each cluster to clonal sizes (**a**) and the shared clonotypes between clusters (**b**). **c** TCR beta chain V- and J-gene usage of CD8$^+$ T cells of ACPA+ RA blood. The width of each band represents the frequency of V- or J- gene usage. ACPA anti-citrullinated protein antibodies, RA rheumatoid arthritis.

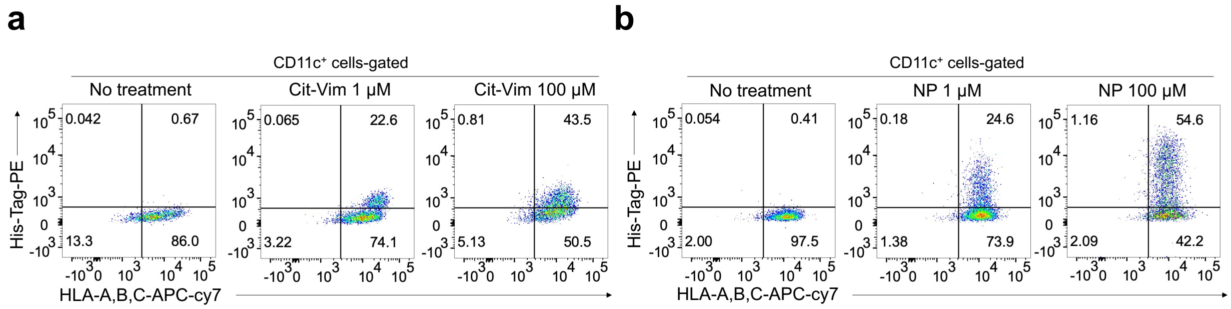

Supplementary Fig. 6: CD11c$^+$ cells from ACPA+ RA patients endocytose cit-vimentin. a,b, Representative flow cytometric plots of protein endocytosis by CD11c$^+$ cells in ACPA+ RA PBMCs show antigen-presenting cells uptake recombinant His-tagged cit-vimentin protein (**a**), or NP protein (**b**) in a dose-dependent manner (1 or 100 μM) after 16 hr of culture. Cit-Vim citrullinated vimentin.

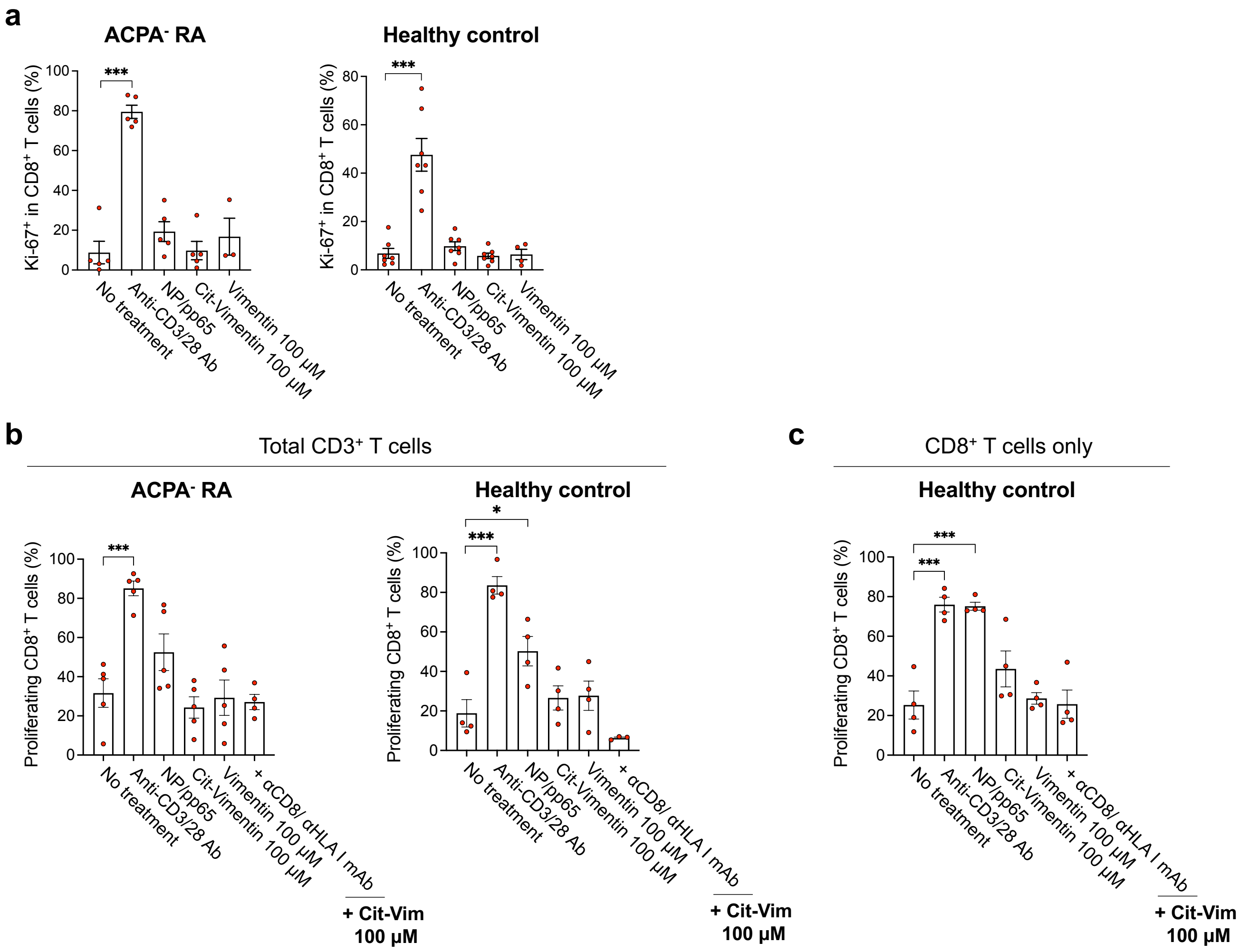

Supplementary Fig. 7: Citrullinated vimentin does not induce the proliferation of ACPA- RA or HC CD8⁺ T cells. a Percentage of Ki-67⁺CD8⁺ T cells in ACPA- RA (*n* = 5) or HC (*n* = 7) PBMCs after stimulation with anti-CD3/28 antibodies, NP (Influenza)/ pp65 (CMV) proteins (50 µM of each), citrullinated vimentin (100 µM), or native vimentin (100 µM) for 16 hr. $^{***}P < 0.0001$. **b, c** Quantification of the proliferating CD8⁺ T cells in co-culture of monocyte-derived dendritic cells (MoDCs) with ACPA- RA or HC total CD3⁺ T cells (**b**, $^{***}P$=0.0002 in ACPA- RA, $^{***}P$=0.0002, or $^{*}P$=0.0214 in HC) or CD8⁺ T cells only (**c**, n = 4, $^{***}P$=0.0007, or $^{***}P$=0.0005). For **b**, no treatment, anti-CD3/28 Ab, NP/pp65, cit-vimentin or native vimentin n = 5, cit-vimentin with anti-CD8/ HLA class I antibodies n = 4 in ACPA- RA and no treatment, anti-CD3/28 Ab, NP/pp65, cit-vimentin or native vimentin n = 4 and cit-vimentin with anti-CD8/ HLA class I antibodies n = 3 in HC. Bars represent means ± SEM. $^{*}P < 0.05$ or $^{***}P < 0.001$ by ordinary one-way ANOVA with Tukey's multiple comparisons test. Source data are provided as

a Source Data file. ACPA anti-citrullinated protein antibodies, RA rheumatoid arthritis. mAb monoclonal antibody.

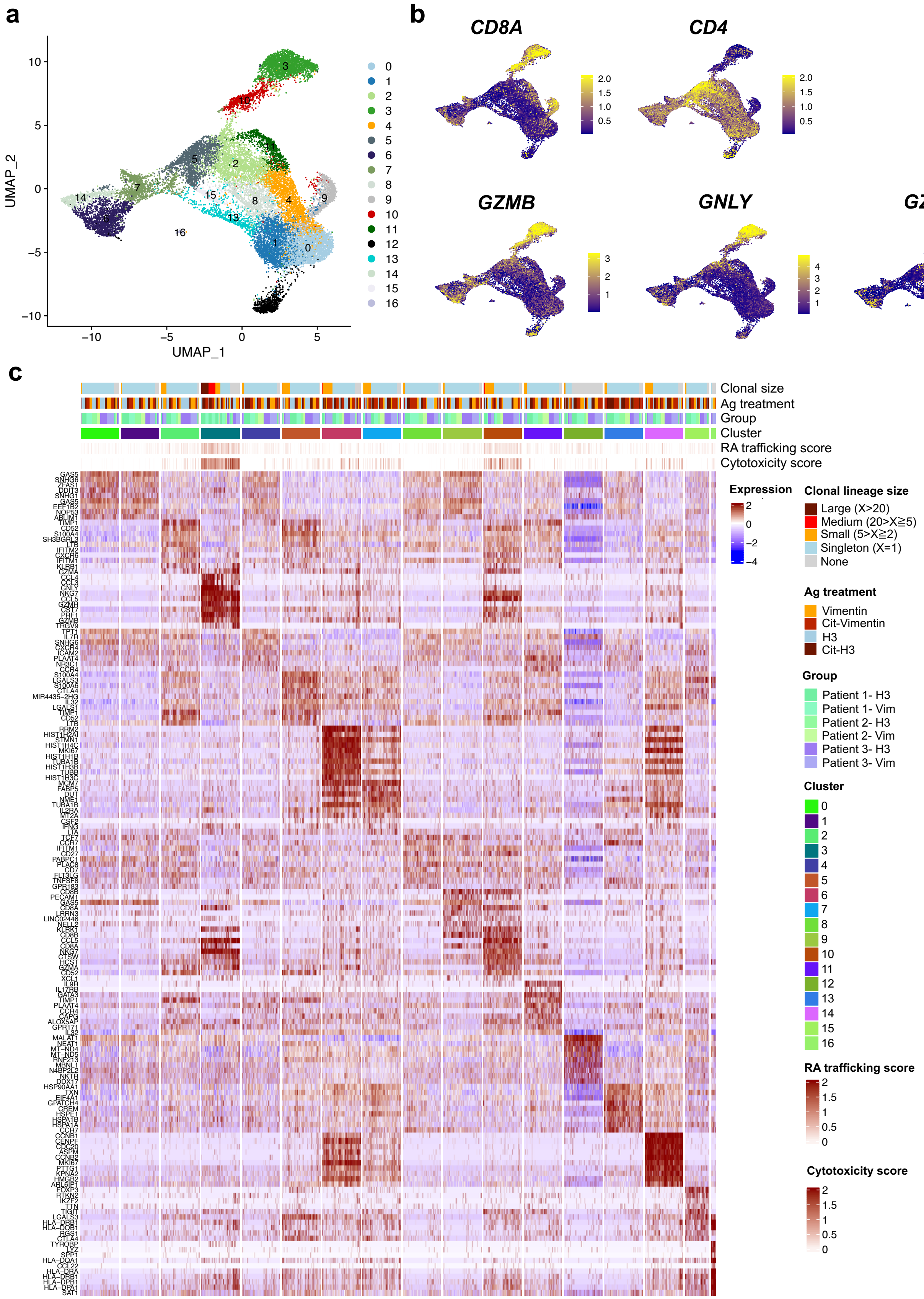
a
UMAP_1
UMAP_2
b
CD8A
CD4
GZMB
GNLY
GZMK
c
Clonal size
Ag treatment
Group
Cluster
RA trafficking score
Cytotoxicity score
Expression
Clonal lineage size
Large (X>20)
Medium (20>X≧5)
Small (5>X≧2)
Singleton (X=1)
None
Ag treatment
Vimentin
Cit-Vimentin
H3
Cit-H3
Group
Patient 1- H3
Patient 1- Vim
Patient 2- H3
Patient 2- Vim
Patient 3- H3
Patient 3- Vim
Cluster
RA trafficking score
Cytotoxicity score

Supplementary Fig. 8: Single-cell RNA sequencing of $CD3^+$ T cells stimulated by native or citrullinated antigens. a UMAP plot representing 17 distinct clusters of $CD3^+$ T cells (n = 13,354) stimulated by native or citrullinated proteins. **b** Expression of *CD8A*, *CD4*, *GZMB*, *GNLY* or *GZMK* in the clusters. Color shows the expression level of each marker. **c** Heatmap showing transcriptional differences in each cluster. Top bars indicate clonal size, Protein treatment, patient group, clusters and scoring scale for RA trafficking and cytotoxicity. Individual T cells are ordered by clonal size in each cluster. RA rheumatoid arthritis, Vim vimentin, Ag antigen.

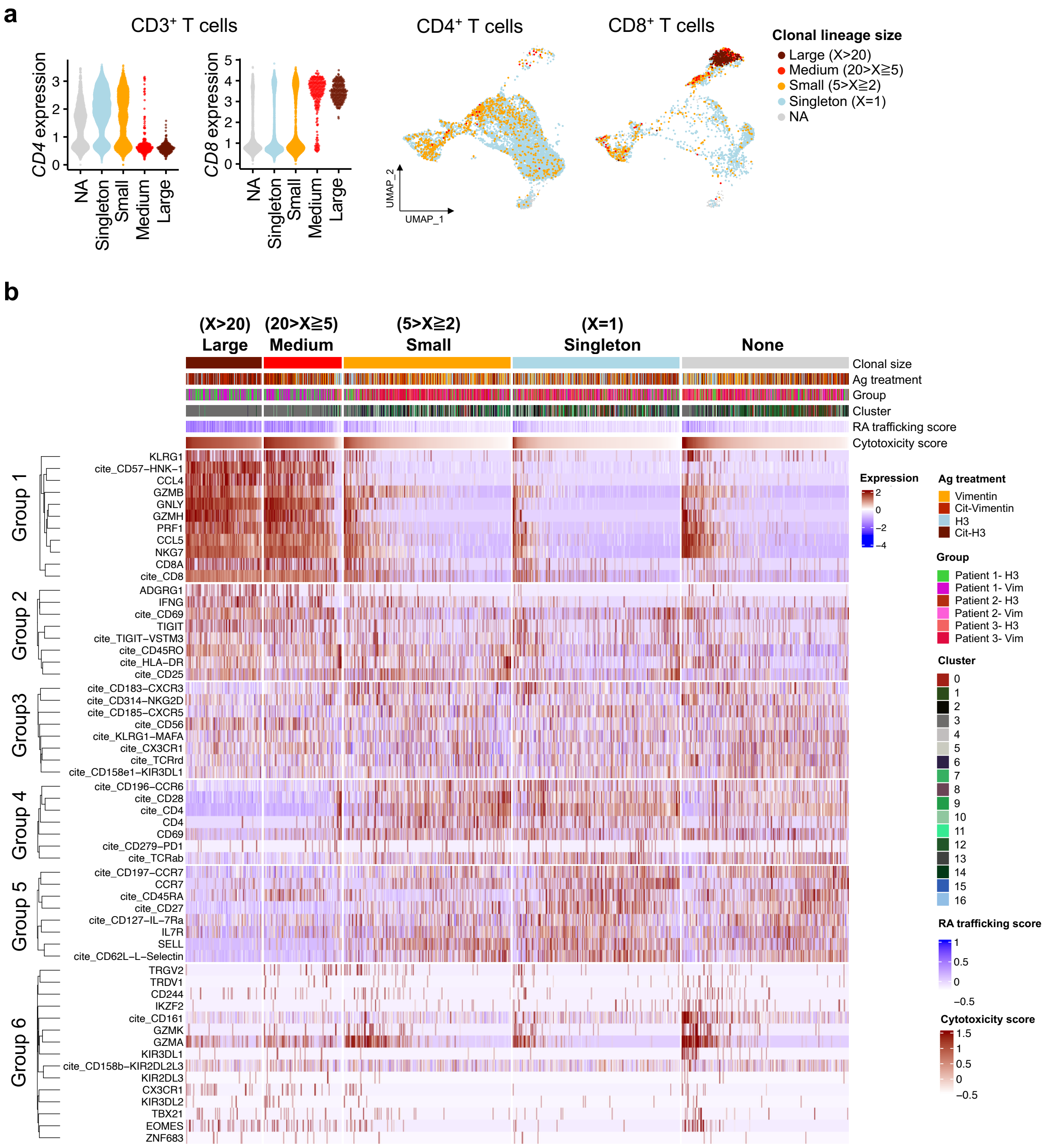

Supplementary Fig. 9: Clonal lineages of CD4$^+$ and CD8$^+$ T cells stimulated by native or citrullinated antigens. a Distribution of each clonal family in *CD4*$^+$ (n = 7,885) or *CD8*$^+$ (n = 3,284) T cells. **b** Heatmap representing 6 groups determined by unsupervised clustering based on the level of RNA and CITE-seq antibodies (cite_). Individual T cells are ordered by cytotoxicity score in each cluster. Ag antigen, Vim vimentin.

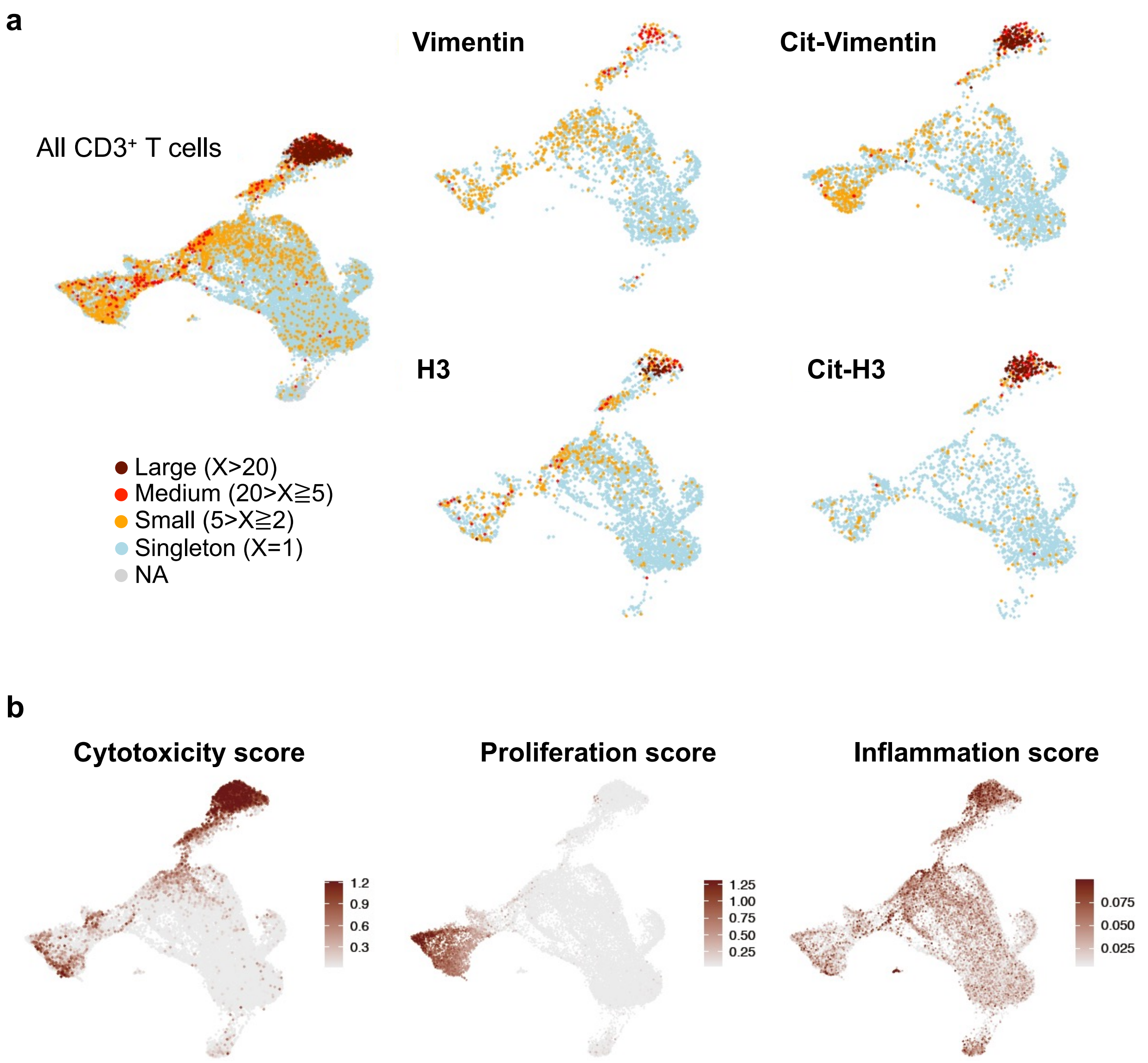

Supplementary Fig. 10: Citrullinated antigens stimulate clonal expansion of CD3$^+$ T cells with high cytotoxicity scores. a UMAP plots of all CD3$^+$ T cells stimulated by native or citrullinated Vimentin or H3 integrated with TCR clonality. **b** UMAP plots showing cytotoxicity, proliferation, or inflammation score. The score was calculated by the expression level of canonical marker genes. Cit- citrullinated.

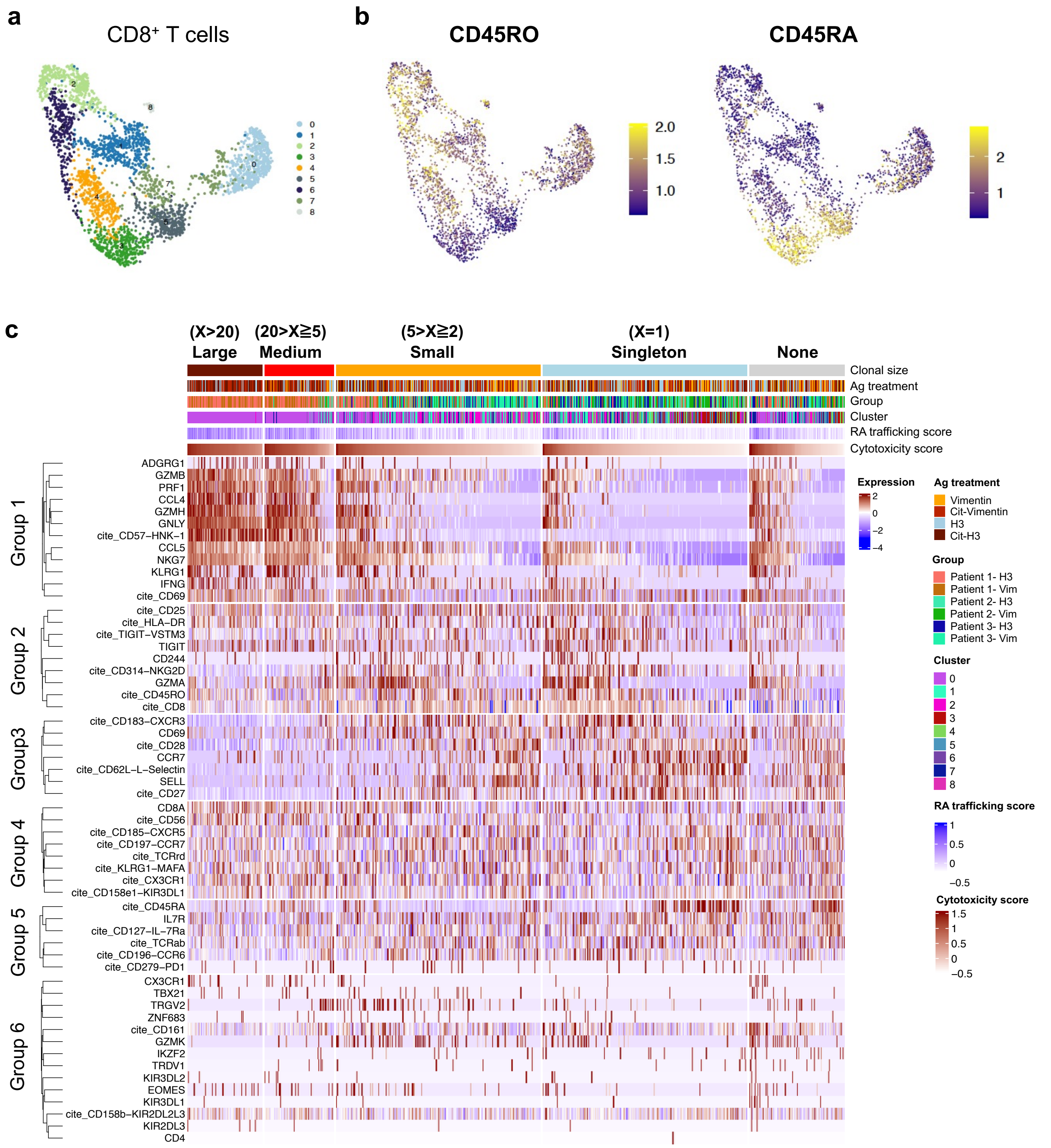

Supplementary Fig. 11: Phenotype of CD8⁺ T cells stimulated by native or citrullinated antigens. a UMAP plot of CD8⁺ T cells having 9 transcriptionally different clusters. **b** Expression level of CD45RO and CD45RA CITE-seq antibodies in CD8⁺ T cells. **c** Heatmap of 6 groups by unsupervised clustering using the level of RNA and CITE-seq antibodies (cite_). RA rheumatoid arthritis, Vim vimentin, Cit-citrullinated.

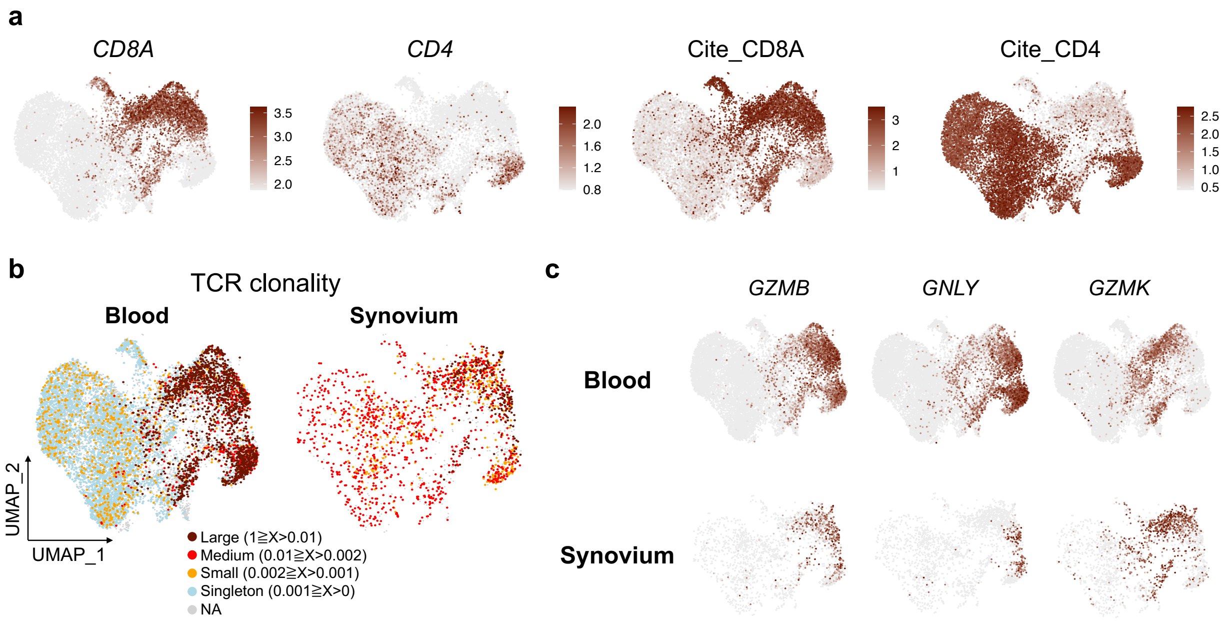

Supplementary Fig. 12: Integrated analysis of single cell RNA-seq data from paired RA blood and synovium CD8$^+$ T cells. a UMAP plots representing the expression levels of CD8A and CD4 in mRNA and CITE-seq Abs. **b** UMAP plots of ACPA+ RA blood (*n* = 3) and synovium (n = 4) CD8$^+$ T cells integrated with TCR clonality (*n* = 9,360 paired *TCRαβ* sequences). Color indicates the groups by the ratio of clonotypes in total cells. **c** UMAP plots showing the expression of *GZMB*, *GNLY* and *GZMK* in integrated blood and synovial CD8$^+$ T cells. Scale bars represent the normalized expression values. TCR T cell receptor.

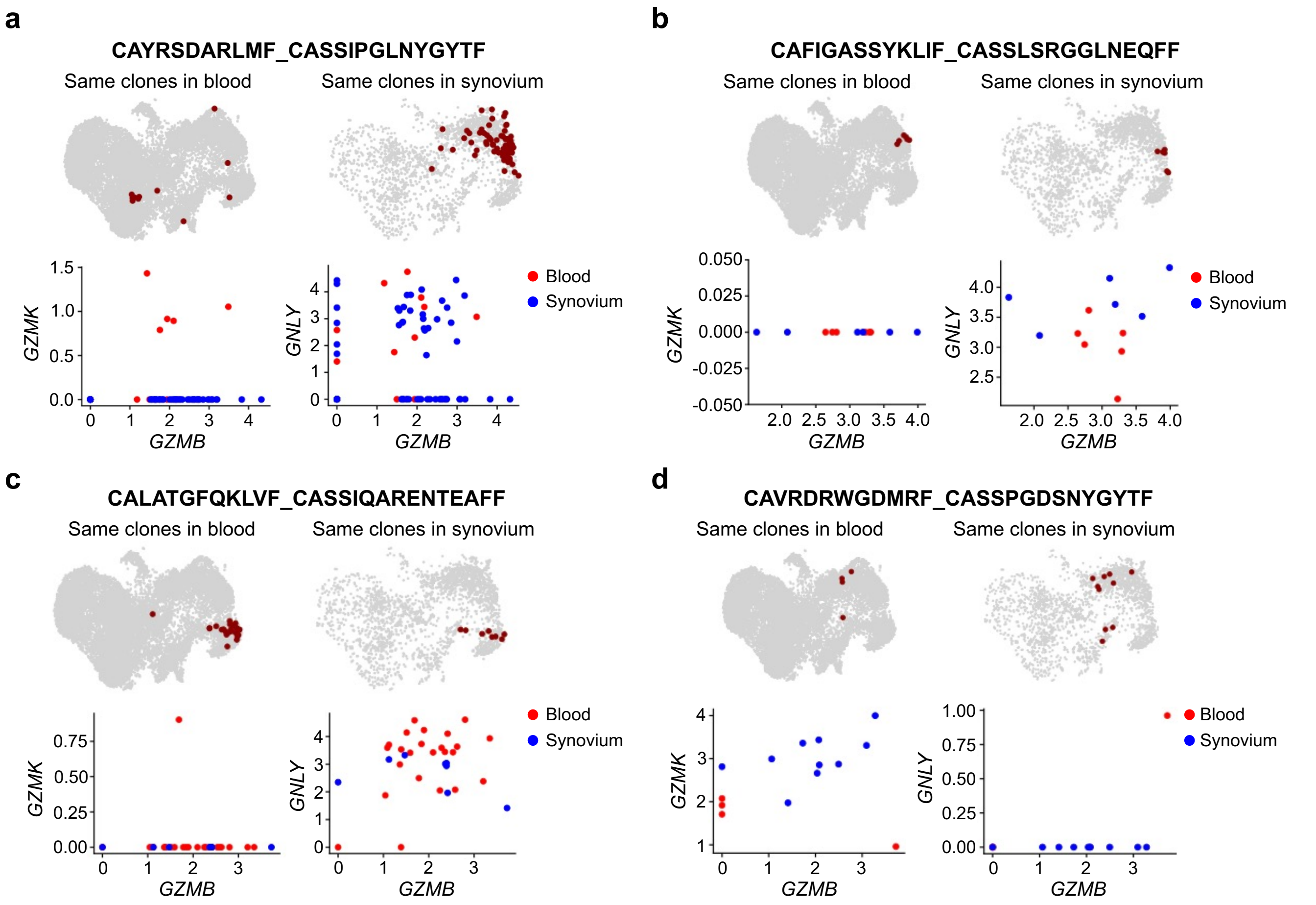

Supplementary Fig. 13: Shared T cell clonotypes between ACPA+ RA blood and matched synovium. a-d Mapping of shared clonotypes in the matched blood and synovium from ACPA+ RA patients. The frequent clonotypes are represented as CDR3 alpha-beta sequences. Dot plots show the expression level of *GZMB*, *GZMK*, and/or *GNLY* in the same clonotypes located in either blood (red) and synovium (blue). Source data are provided as a Source Data file.

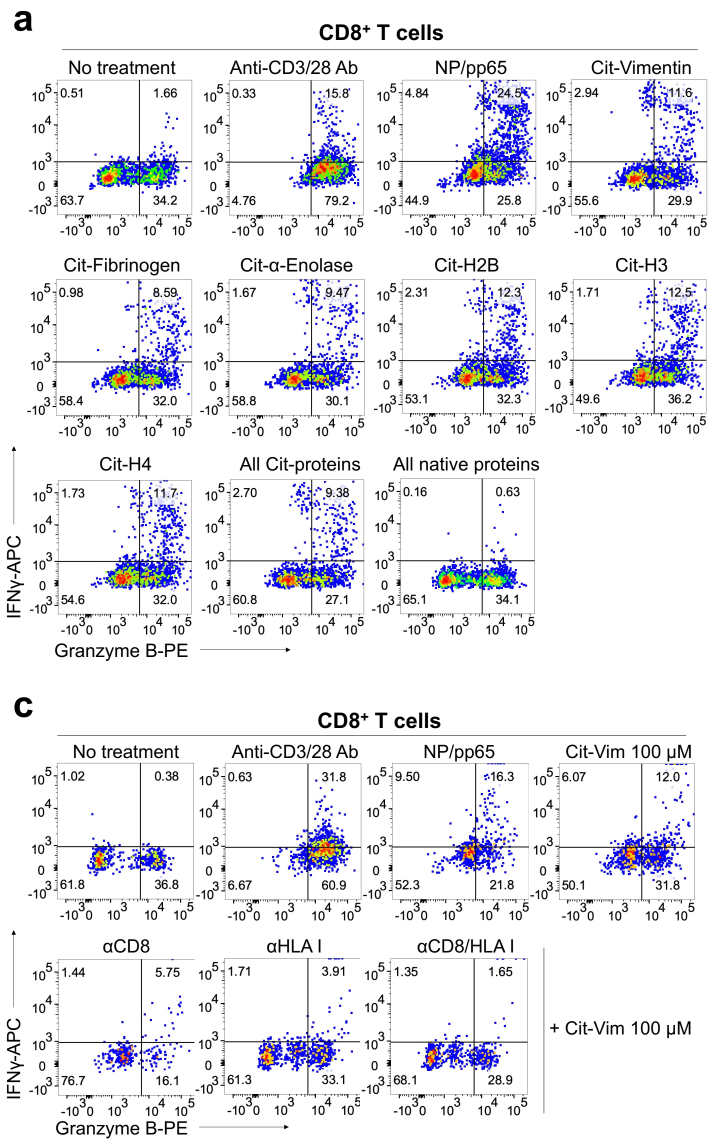

Supplementary Fig. 14: ACPA+ RA patient blood CD8^{+} T cells are activated by citrullinated proteins in a CD8:HLA class I-dependent manner. a-c Representative flow cytometric plots of ACPA+ RA blood CD8^{+} T cells stimulated with citrullinated proteins (**a**), a range of concentrations of cit-vimentin or native vimentin (**b**), and in the presence of anti-CD8/ HLA class I-blocking antibodies (**c**). Ab antibody, Vim vimentin, Cit- citrullinated.

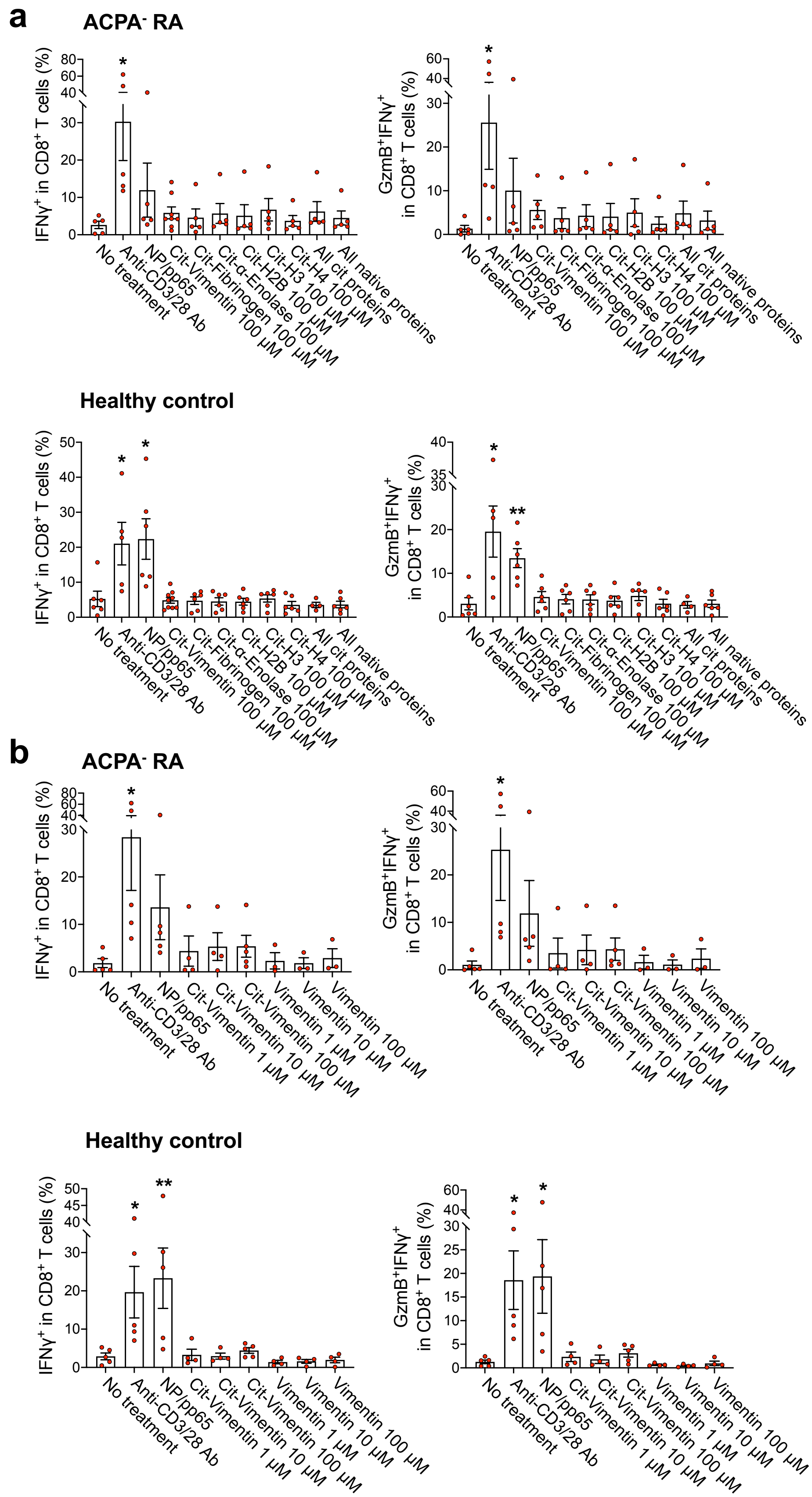
a
ACPA- RA
IFNγ+ in CD8+ T cells (%)
GzmB+IFNγ+ in CD8+ T cells (%)
No treatment
Anti-CD3/28 Ab
NP/pp65
Cit-Vimentin 100 μM
Cit-Fibrinogen 100 μM
Cit-α-Enolase 100 μM
Cit-H2B 100 μM
Cit-H3 100 μM
Cit-H4 100 μM
All cit proteins
All native proteins
Healthy control
b
ACPA- RA
Cit-Vimentin 1 μM
Cit-Vimentin 10 μM
Cit-Vimentin 100 μM
Vimentin 1 μM
Vimentin 10 μM
Vimentin 100 μM
Healthy control

Supplementary Fig. 15: ACPA- RA patient $CD8^+$ T cells react with viral but not citrullinated antigens. a Percentages of IFNγ and/ or GzmB-expressing $CD8^+$ T cells from PBMCs of ACPA- RA or HCs were measured by intracellular staining after stimulation with anti-CD3/28 antibodies, NP (Influenza)/ pp65 (CMV) proteins (50 μM of each), citrullinated proteins (vimentin, fibrinogen, α-enolase, and histones H2B, H3 or H4) (100 μM), all citrullinated proteins (20 μM each), or all native proteins (20 μM each) for 16 hr. For ACPA- RA, no stimulation (n = 5), anti-CD3/28 Abs (n = 5), NP/pp65 (n = 5), cit-vimentin ($IFN\gamma^+$ n = 8, $GzmB^+IFN\gamma^+$ n = 5), cit-fibrinogen, α-enolase, and histones H2B, H3 or H4 (n = 5), all cit proteins (n = 5), or all native proteins (n = 5); $^*P$=0.0296, or $^*P$=0.0498. For HC, no stimulation (n = 6), anti-CD3/28 Abs (n = 5), NP/pp65 (n = 6), cit-vimentin ($IFN\gamma^+$ n = 9, $GzmB^+IFN\gamma^+$ n = 6), cit-fibrinogen, α-enolase, and histones H2B, H3 or H4 (n = 6), all cit proteins (n = 4), or all native proteins (n = 6); $^*P$=0.0274, $^*P$=0.0201, $^*P$=0.0150, or $^{**}P$=0.0023. **b** Quantification of $IFN\gamma^+$ or $IFN\gamma^+GzmB^+$ $CD8^+$ T cells in ACPA- RA or HC PBMCs stimulated for 16 hr with cit-vimentin or native vimentin in a concentration-dependent manner. No stimulation (ACPA- RA and HC n = 5), anti-CD3/28 Abs (ACPA- RA and HC n = 5), NP/pp65 (ACPA- RA and HC n = 5), cit-vimentin 1 or 10 μM (ACPA- RA and HC n = 4), cit-vimentin 100 μM (ACPA- RA and HC n = 5), vimentin 1,10 or 100 μM (ACPA- RA n = 3 and HC n = 4). In ACPA- RA, $^*P$=0.0462, or $^*P$=0.05. In HC, $^*P$=0.0385, $^{**}P$=0.033, $^*P$=0.024, or $^*P$=0.0491. For **a, b,** data are presented as means ± SEM. By two-tailed unpaired *t*-test: $^*P < 0.05$ or $^{**}P < 0.01$ versus no treatment. Source data are provided as a Source Data file. ACPA anti-citrullinated protein antibodies, RA rheumatoid arthritis, Ab antibody.

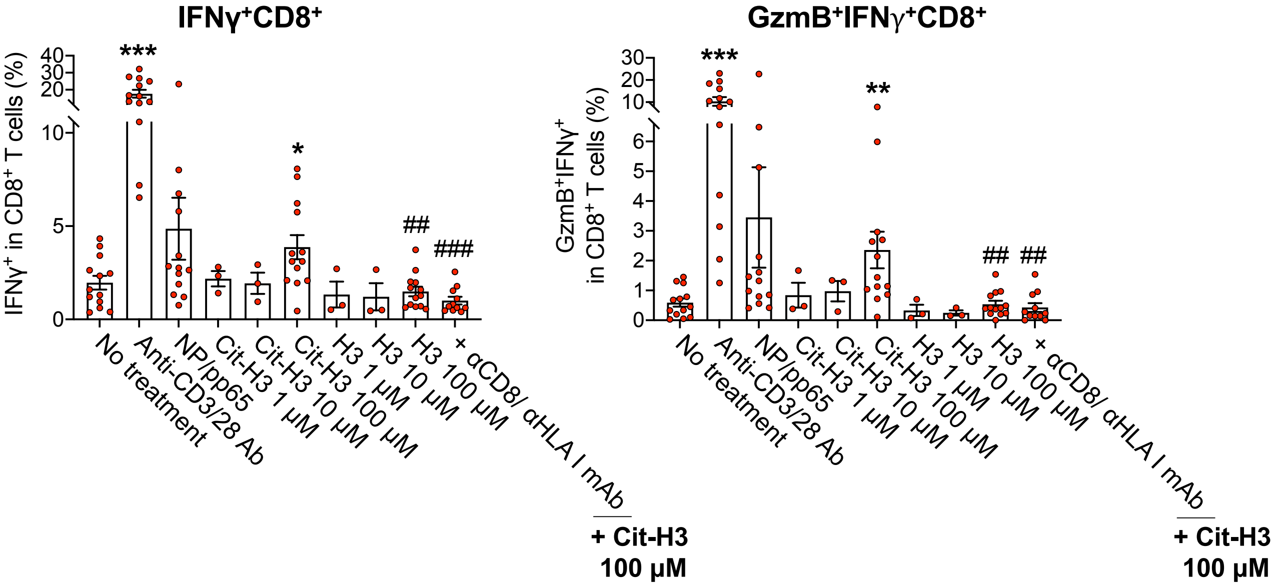

Supplementary Fig. 16: ACPA+ RA blood CD8$^+$ T cells react with cit-H3.

Percentage of IFNγ or GzmB expressing CD8$^+$ T cells in ACPA+ RA blood measured by intracellular staining after stimulation with a range of concentrations of citrullinated histone H3 (cit-H3) or native H3 protein in presence or absence of anti-CD8/HLA class I-blocking antibodies for 16 hrs. No stimulation (n = 13), anti-CD3/28 Abs (n = 13), NP/pp65 (n = 13), cit-H3 or native H3 1 or 10 μM (n = 3), cit-H3 or native H3 100 μM (n = 13), cit-H3 with anti-CD8/ HLA class I antibodies (n = 11). For IFNγ$^+$CD8$^+$, $^{***}P$<0.0001, $^{*}P$=0.0172, $^{\#\#}P$=0.0023, or $^{\#\#\#}P$=0.0007. For GzmB$^+$IFNγ$^+$CD8$^+$, $^{***}P$<0.0001, $^{**}P$=0.0098, $^{\#\#}P$=0.0077, or $^{\#\#}P$=0.0099. Data are presented as means ± SEM. By two-tailed unpaired *t*-test: *P < 0.05, **P < 0.01 or ***P < 0.001 versus no treatment, and $^{\#\#}P$ < 0.01 or $^{\#\#\#}P$ < 0.001 versus cit-H3 100 μM. Source data are provided as a Source Data file. Ab antibody.

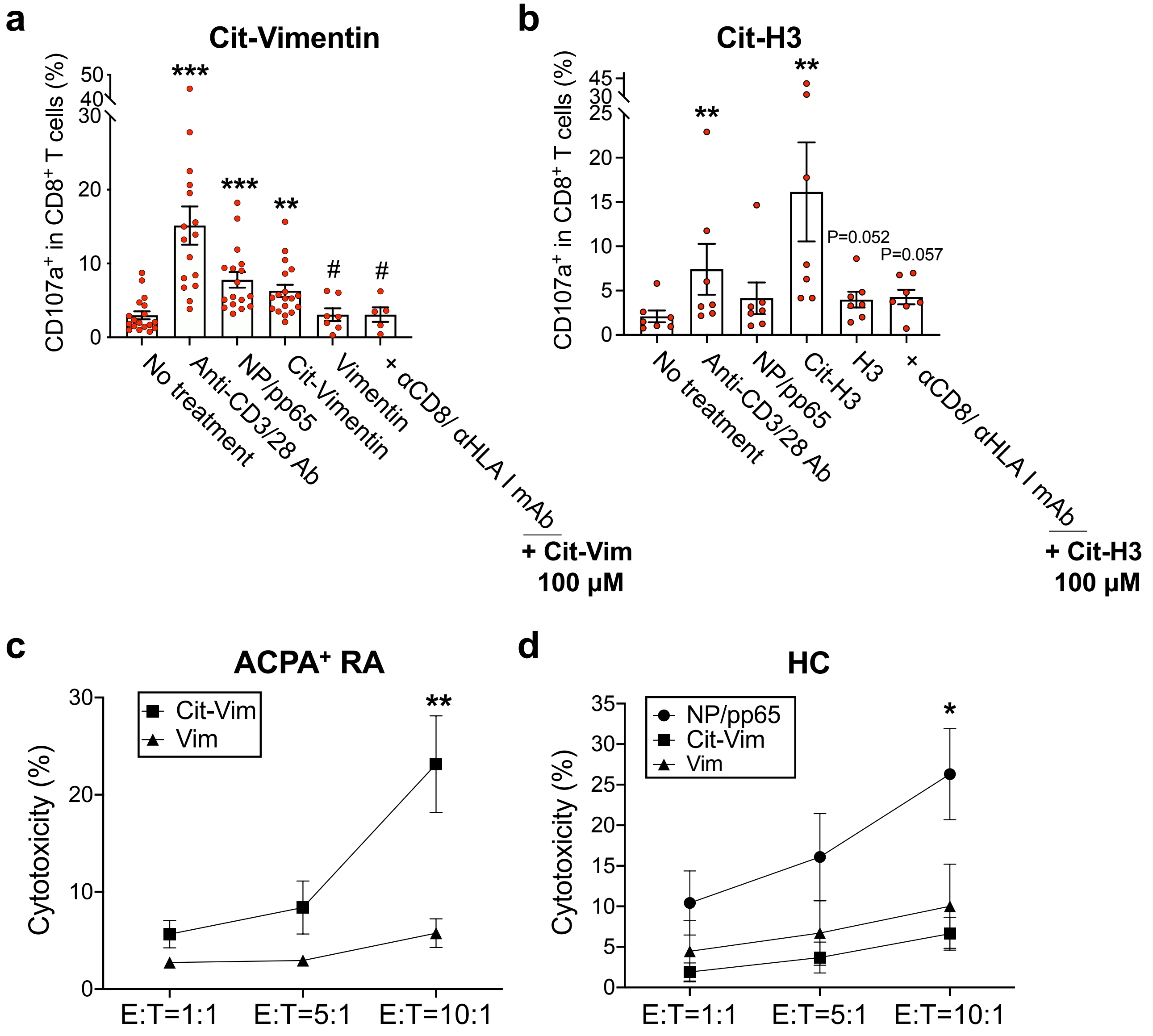

Supplementary Fig. 17: Citrullinated antigens stimulate ACPA+ RA blood CD8$^+$ T cells to exhibit cytotoxic activity and mediate cell killing. a, b Flow cytometry analysis of CD107a$^+$ expression by CD8$^+$ T cells was used as a measure of degranulation activity. ACPA+ RA blood samples were stimulated with citrullinated or native forms of the RA autoantigens vimentin and histone H3 (100 µM each), with or without anti-CD8/ HLA class I-blocking antibodies, and flow cytometry analysis performed to quantitate CD107a$^+$CD8$^+$ T cells. For (**a**), no stimulation (n = 18), anti-CD3/28 Abs (n = 16), NP/pp65 (n = 17), cit-vimentin (n = 18), vimentin (n = 7), cit-vimentin with anti-CD8/ HLA class I antibodies (n = 5); $^{***}P < 0.0001$, $^{***}P$=0.0003, $^{**}P$=0.0021, $^{\#}P$=0.0364, or $^{\#}P$=0.0494. For (**b**), n = 7; $^{***}P < 0.0001$, or $^{*}P$=0.028. **c, d** Antigen-independent cytotoxic activity of cit-vimentin, native vimentin, or viral protein

stimulated $CD8^+$ T cells from ACPA+ RA (**c**) or HC (**d**) blood against the DLD-1 cell line was determined using a lactate dehydrogenase (LDH) release assay. For (**c**), cit-vimentin (n = 7) and vimentin (n = 5); $^{**}P$=0.0025. For (**d**), NP/pp65 (n = 14), cit-vimentin or vimentin (n = 3). E:T ratio means effector/target cell ratio. Data are presented as means ± SEM. For (**a, b**), using two-tailed unpaired *t*-test: $^{*}P < 0.05$, $^{**}P < 0.01$, or $^{***}P < 0.001$ versus no treatment; $^{\#}P < 0.05$ versus cit-vimentin; *P* value represented on the graph was compared with cit-vimentin. For (**c, d**), using two-way ANOVA with Tukey's multiple comparisons test: $^{**}P < 0.01$. Source data are provided as a Source Data file. HC healthy control, ACPA anti-citrullinated protein antibodies, RA rheumatoid arthritis, mAb monoclonal antibody, Cit-Vim citrullinated vimentin.

Supplementary Tables

Supplementary Table 1. Demographic characteristics of RA patients.

	**RA patients**
Total Number	59
Gender, Female/Male	4/55
Age (years) - mean±SD	66.15±12.24
Dis duration (years) - mean±SD	11.35±10.69
Disease activity	
ACPA positive (%)	76.27
RF positive (%)	73.33
CDAI score - mean±SD	15.17±15.95
MTX use (%)	51.11
MTX duration (years)	4.96±4.9

Supplementary Table 2 Differentially expressed genes in each cluster identified in ACPA+ RA as compared to HC blood. Differentially expressed genes in each cluster were selected with Log2 Fold change > 0.8. Wilcoxon Rank Sum test and the Benjamini–Hochberg method to adjust the *P* values for multiple testing. Avg average, Pct percentage, p_val_adj adjusted *P* value.

Gene	Avg_log2FC	Pct.1	Pct.2	p_val_adj	Cluster
CCR7	2.17188736	0.952	0.528	0	Naïve
LEF1	2.00017763	0.954	0.652	0	Naïve
ACTN1	1.50463023	0.94	0.711	0	Naïve
NELL2	1.42062488	0.889	0.612	0	Naïve
MAL	1.32861183	0.804	0.643	0	Naïve
LTB	1.29286009	0.964	0.78	0	Naïve
SELL	1.25489209	0.951	0.817	0	Naïve
NOSIP	1.2525314	0.947	0.812	0	Naïve
OXNAD1	1.2385171	0.956	0.681	0	Naïve
PIK3IP1	1.1523528	0.96	0.805	0	Naïve
AIF1	1.14401643	0.867	0.767	0	Naïve
TRABD2A	1.07210543	0.915	0.757	0	Naïve
LDLRAP1	1.05680921	0.928	0.822	0	Naïve
CD27	1.03820024	0.934	0.718	0	Naïve
SPINT2	1.02349056	0.967	0.724	0	Naïve
SNHG8	0.99485116	0.993	0.924	0	Naïve
NDFIP1	0.97624462	0.986	0.869	0	Naïve
ABLIM1	0.96524899	0.787	0.402	0	Naïve
CD8B	0.90637784	0.98	0.9	0	Naïve
CAMK4	0.85677228	0.958	0.737	0	Naïve
ARMH1	0.82194778	0.793	0.738	0	Naïve
HSPB1	0.79107008	0.972	0.821	0	Naïve
PCED1B	0.76851258	0.867	0.737	0	Naïve
RCAN3	0.74887121	0.782	0.717	0	Naïve
LDHB	0.74089477	0.961	0.907	0	Naïve
TCF7	0.95014837	0.841	0.758	3.76E-301	Naïve
CD55	0.86560341	0.88	0.786	3.17E-286	Naïve
SATB1	0.76575809	0.749	0.593	1.77E-205	Naïve
FLT3LG	0.71486197	0.879	0.791	1.39E-199	Naïve
S100B	0.81117364	0.72	0.75	1.09E-184	Naïve

FGFBP2	2.64092332	0.994	0.896	0	*GZMB+ GNLY+*
GZMH	2.48081498	0.982	0.899	0	*GZMB+ GNLY+*
GNLY	2.2873411	0.956	0.666	0	*GZMB+ GNLY+*
GZMB	2.20826241	0.949	0.829	0	*GZMB+ GNLY+*
ZNF683	1.93895227	0.954	0.888	0	*GZMB+ GNLY+*
NKG7	1.86436384	0.999	0.888	0	*GZMB+ GNLY+*
PRF1	1.71556813	0.988	0.773	0	*GZMB+ GNLY+*
ADGRG1	1.69693841	0.956	0.796	0	*GZMB+ GNLY+*
LGALS1	1.62412354	0.905	0.789	0	*GZMB+ GNLY+*
SPON2	1.56725113	0.933	0.694	0	*GZMB+ GNLY+*
PRSS23	1.46520941	0.945	0.865	0	*GZMB+ GNLY+*
FCGR3A	1.40710564	0.927	0.738	0	*GZMB+ GNLY+*
CX3CR1	1.31747937	0.874	0.788	0	*GZMB+ GNLY+*
ZEB2	1.29236084	0.834	0.797	0	*GZMB+ GNLY+*
PLEK	1.27834466	0.87	0.707	0	*GZMB+ GNLY+*
CST7	1.25537403	0.993	0.952	0	*GZMB+ GNLY+*
CCL4	1.25062717	0.856	0.876	0	*GZMB+ GNLY+*
ITGB1	1.21597628	0.94	0.911	0	*GZMB+ GNLY+*
KLRD1	1.21207058	0.915	0.787	0	*GZMB+ GNLY+*
EFHD2	1.17334611	0.876	0.717	0	*GZMB+ GNLY+*
FCRL6	1.15172947	0.935	0.93	0	*GZMB+ GNLY+*
TBX21	1.15137019	0.938	0.891	0	*GZMB+ GNLY+*
GZMA	1.14945792	0.986	0.847	0	*GZMB+ GNLY+*
S100A4	1.13366146	0.995	0.937	0	*GZMB+ GNLY+*
ITGB2	1.07810461	0.995	0.959	0	*GZMB+ GNLY+*
FLNA	1.06917519	0.974	0.941	0	*GZMB+ GNLY+*
S1PR5	1.01688766	0.906	0.847	0	*GZMB+ GNLY+*
SLC9A3R1	0.98919573	0.974	0.894	0	*GZMB+ GNLY+*
CCL5	0.97731527	0.994	0.97	0	*GZMB+ GNLY+*
HOPX	0.97559354	0.913	0.871	0	*GZMB+ GNLY+*
FCGR3A	1.78521332	0.98	0.784	3.47E-253	*GZMB+ KIR+*
TTC38	1.06213688	0.961	0.761	3.68E-205	*GZMB+ KIR+*
KIR3DL2	0.78538508	0.816	0.57	4.05E-177	*GZMB+ KIR+*
TRDV1	1.75850042	0.823	0.758	3.73E-166	*GZMB+ KIR+*
NKG7	1.08540583	0.999	0.916	6.13E-161	*GZMB+ KIR+*
S1PR5	0.96269525	0.953	0.86	1.03E-154	*GZMB+ KIR+*
GZMB	1.12077486	0.971	0.859	1.27E-147	*GZMB+ KIR+*
KLRC3	1.17386824	0.793	0.594	2.35E-140	*GZMB+ KIR+*
ADGRG1	0.93451484	0.941	0.837	1.72E-135	*GZMB+ KIR+*

CST7	0.93584796	0.991	0.962	1.76E-130	*GZMB+ KIR+*
PRF1	1.01856434	0.989	0.828	4.97E-124	*GZMB+ KIR+*
TBX21	0.78939386	0.944	0.903	1.23E-123	*GZMB+ KIR+*
C1orf21	0.7426384	0.904	0.855	7.30E-122	*GZMB+ KIR+*
KLRD1	1.06721614	0.936	0.819	3.51E-117	*GZMB+ KIR+*
FGR	0.87854003	0.909	0.886	2.99E-115	*GZMB+ KIR+*
FCRL6	0.8177497	0.964	0.93	9.00E-114	*GZMB+ KIR+*
FGFBP2	0.8305155	0.983	0.922	9.59E-108	*GZMB+ KIR+*
CCL5	0.78234757	0.995	0.976	9.86E-99	*GZMB+ KIR+*
PLEK	0.84156567	0.912	0.747	7.33E-93	*GZMB+ KIR+*
GZMH	0.76278171	0.967	0.921	9.21E-89	*GZMB+ KIR+*
CMC1	0.83244954	0.816	0.632	8.14E-75	*GZMB+ KIR+*
CCL4	0.8534083	0.916	0.868	2.86E-68	*GZMB+ KIR+*
IKZF2	0.77393067	0.693	0.574	4.91E-65	*GZMB+ KIR+*
TIGIT	1.00714799	0.741	0.696	9.89E-58	*GZMB+ KIR+*
ZEB2	0.80594977	0.82	0.807	3.56E-55	*GZMB+ KIR+*
CD63	0.7813272	0.859	0.812	1.37E-53	*GZMB+ KIR+*
GNLY	0.80162342	0.881	0.744	2.43E-53	*GZMB+ KIR+*
KLRF1	0.93276105	0.648	0.531	5.53E-38	*GZMB+ KIR+*
NCR1	0.82343017	0.592	0.417	5.63E-19	*GZMB+ KIR+*
TYROBP	0.84125194	0.66	0.688	3.20E-12	*GZMB+ KIR+*
TRDV2	1.99128693	0.913	0.407	0	*TCRgd+*
TRGV9	1.92133086	0.852	0.666	1.24E-221	*TCRgd+*
TYROBP	1.33879925	0.875	0.677	2.75E-189	*TCRgd+*
MAP3K8	0.63507239	0.974	0.865	1.50E-171	*TCRgd+*
S100B	0.62382483	0.867	0.736	5.13E-162	*TCRgd+*
DUSP2	1.05992088	0.996	0.965	4.05E-146	*TCRgd+*
KLRB1	1.04734785	0.882	0.763	1.68E-121	*TCRgd+*
MATK	0.71312879	0.949	0.881	6.32E-108	*TCRgd+*
KLRG1	0.85459333	0.913	0.73	2.58E-103	*TCRgd+*
KLRD1	0.77247908	0.933	0.819	1.04E-102	*TCRgd+*
ID2	0.92916918	0.965	0.78	3.99E-97	*TCRgd+*
ALOX5AP	0.80492512	0.915	0.815	1.02E-71	*TCRgd+*
GNLY	0.74442669	0.879	0.743	5.58E-71	*TCRgd+*
CST7	0.58130969	0.99	0.962	1.93E-64	*TCRgd+*
NKG7	0.57142342	0.998	0.916	3.11E-63	*TCRgd+*
DUSP1	0.82391136	0.98	0.889	2.01E-61	*TCRgd+*
KRT10	0.55492149	0.988	0.941	6.52E-59	*TCRgd+*
TRDC	1.1402084	0.663	0.562	4.61E-51	*TCRgd+*

PRELID1	0.56047116	0.969	0.858	2.82E-46	*TCRgd+*
ANXA1	0.52941704	0.973	0.894	4.07E-45	*TCRgd+*
APOBEC3G	0.53168543	0.853	0.825	6.76E-42	*TCRgd+*
ZFP36	0.52644306	0.981	0.916	3.04E-38	*TCRgd+*
IL2RB	0.53117476	0.928	0.861	1.33E-31	*TCRgd+*
CD300A	0.51766821	0.7	0.544	1.28E-24	*TCRgd+*
NCR3	0.5182753	0.65	0.648	2.80E-12	*TCRgd+*
TRGC1	0.68535161	0.565	0.393	4.71E-10	*TCRgd+*
KLRC1	0.58682728	0.394	0.33	7.14E-06	*TCRgd+*
GZMK	1.99717379	0.895	0.768	0	*GZMK+*
DUSP2	1.35848465	0.991	0.96	0	*GZMK+*
CD74	1.07464734	0.946	0.907	0	*GZMK+*
COTL1	0.66638748	0.93	0.872	7.40E-248	*GZMK+*
RGS1	0.83275317	0.705	0.643	9.78E-207	*GZMK+*
FYN	0.81771733	0.904	0.784	1.04E-193	*GZMK+*
CMC1	1.0803568	0.754	0.615	1.22E-184	*GZMK+*
CCL4	0.63132847	0.892	0.866	3.33E-149	*GZMK+*
CCL5	0.52091684	0.989	0.975	5.05E-143	*GZMK+*
LYAR	0.58186799	0.882	0.839	3.17E-104	*GZMK+*
IL10RA	0.51340213	0.945	0.897	4.31E-87	*GZMK+*
TIGIT	0.68709491	0.686	0.701	1.23E-81	*GZMK+*
LYST	0.56801848	0.693	0.615	2.91E-77	*GZMK+*
PIK3R1	0.57399294	0.74	0.748	1.04E-46	*GZMK+*
LTB	1.07119384	0.977	0.804	0	Memory
IL7R	1.03381631	0.981	0.791	0	Memory
COTL1	0.76448942	0.959	0.871	0	Memory
CAPG	0.55036677	0.898	0.714	0	Memory
AQP3	0.54135708	0.898	0.798	5.71E-169	Memory
LGALS3	0.58233773	0.816	0.783	2.94E-168	Memory
JUNB	0.65509324	0.997	0.977	3.32E-153	Memory
ARHGAP15	0.76563726	0.913	0.809	2.03E-151	Memory
PTGER2	0.55948452	0.912	0.815	5.78E-145	Memory
LDHB	0.60480366	0.963	0.915	1.60E-143	Memory
CXCR3	0.64707736	0.868	0.738	4.90E-143	Memory
FXYD2	0.72697369	0.762	0.702	1.39E-142	Memory
FOS	0.59514237	0.921	0.784	6.91E-139	Memory
TIMP1	0.619823	0.838	0.698	3.64E-115	Memory
PLP2	0.68495292	0.86	0.759	9.64E-105	Memory
TCF7	0.53829795	0.884	0.763	1.00E-100	Memory

KLRB1	2.46140509	0.967	0.757	0	*CCR6+ CD161+*
LST1	0.65603783	0.865	0.596	1.93E-208	*CCR6+ CD161+*
IL7R	1.23562461	0.984	0.806	4.41E-207	*CCR6+ CD161+*
DPP4	0.91774681	0.888	0.701	1.12E-188	*CCR6+ CD161+*
TYROBP	1.19564119	0.884	0.675	1.58E-184	*CCR6+ CD161+*
MYC	0.7221775	0.942	0.667	4.50E-178	*CCR6+ CD161+*
TTC39C	0.78485329	0.964	0.854	2.13E-173	*CCR6+ CD161+*
GZMK	1.10947754	0.925	0.783	1.66E-162	*CCR6+ CD161+*
NFKBIA	1.35943949	0.936	0.831	2.11E-147	*CCR6+ CD161+*
NCR3	1.36753518	0.838	0.637	1.26E-143	*CCR6+ CD161+*
DUSP1	1.12077918	0.982	0.888	3.22E-142	*CCR6+ CD161+*
AQP3	1.08914582	0.927	0.804	1.39E-133	*CCR6+ CD161+*
LGALS3	0.95770062	0.805	0.786	3.32E-125	*CCR6+ CD161+*
SLAMF1	0.66166051	0.811	0.609	1.59E-108	*CCR6+ CD161+*
ALOX5AP	0.97222512	0.922	0.813	6.24E-106	*CCR6+ CD161+*
GCHFR	0.84474297	0.805	0.628	2.54E-105	*CCR6+ CD161+*
KLRG1	0.91738742	0.904	0.73	1.29E-93	*CCR6+ CD161+*
CXCR6	0.89331155	0.719	0.547	7.54E-90	*CCR6+ CD161+*
CCR6	0.81160558	0.728	0.758	1.09E-88	*CCR6+ CD161+*
LTB	0.80620585	0.967	0.817	1.07E-75	*CCR6+ CD161+*
ZBTB16	0.90784986	0.697	0.641	6.60E-71	*CCR6+ CD161+*
CD69	0.83337076	0.925	0.84	3.02E-52	*CCR6+ CD161+*
GBP5	0.60826196	0.851	0.675	5.81E-48	*CCR6+ CD161+*
FOS	0.65314205	0.928	0.794	1.06E-43	*CCR6+ CD161+*
ERN1	0.83350715	0.73	0.697	4.33E-36	*CCR6+ CD161+*
SLC4A10	0.89645098	0.615	0.378	8.03E-31	*CCR6+ CD161+*
JAML	0.6162617	0.669	0.581	7.01E-29	*CCR6+ CD161+*
ZFP36L1	0.69958413	0.82	0.735	5.53E-26	*CCR6+ CD161+*
TMIGD2	0.72239382	0.697	0.714	9.21E-26	*CCR6+ CD161+*
DDIT4	0.7047064	0.984	0.947	1.59E-17	*CCR6+ CD161+*

Supplementary Table 3. HLA typing of ACPA+ RA patients. 12 ACPA+ RA patients were randomly selected and sequenced to get HLA types.

Sample ID	A/1	A/2	B/1	B/2	C/1	C/2
**JG330**	A*29:02:01:01	A*32:01:01:01	B*35:03:01:01	B*44:02:01:01	C*12:03:01:01	C*05:01:01:02
**JG331**	A*24:07:01	A*11:02:01	B*15:35	B*55:02:01:03	C*07:02:01:01	C*01:02:01:01
**JG332**	A*24:02:01:01	A*01:01:01:01	B*27:05:02:05	B*08:01:01:01	C*02:02:02:01	C*07:01:01:01
**JG127**	A*03:01:01:01	A*01:01:01:01	B*35:01:01:05	B*52:01:01:02	C*04:01:01:11	C*12:02:02:01
**JG128**	A*23:01:01:01	A*11:01:01:01	B*07:02:01:01	B*35:01:01:05	C*07:02:01:03	C*04:01:01:01
**JG129**	A*23:01:01:01	A*68:01:01:02	B*49:01:01:01	B*15:220:01:01	C*07:01:01:16	C*04:01:01:01
**JG143**	A*02:01:01:01	A*74:01:01:01	B*42:01:01	B*27:05:02:01	C*17:01:01:02	C*15:02:01:01
**JG147**	A*11:01:01:01	A*02:06:01:01	B*40:01:02:01	B*51:01:01:05	C*03:04:01:02	C*15:02:01:03
**JG149**	A*24:02:01:01	A*31:01:02:01	B*08:01:01:01	B*57:01:01:01	C*07:01:01:01	C*06:02:01:01
**JG159**	A*29:02:01:02	A*29:02:01:01	B*45:01:01:01	B*44:03:01:01	C*06:02:01:01	C*16:01:01:01
**JG262**	A*03:01:01:01	A*01:01:01:01	B*40:01:02:01	B*08:01:01:01	C*03:04:01:01	C*07:01:01:01
**JG272**	A*30:01:01:01	A*30:01:01:01	B*53:01:01:01	B*44:03:01:10	C*04:01:01:14	C*17:01:01:02
**Sample ID**	**DPA1/1**	**DPA1/2**	**DPB1/1**	**DPB1/2**	**DQA1/1**	**DQA1/2**
**JG330**	DPA1*02:02:02:01	DPA1*01:03:01:05	DPB1*05:01:01:01	DPB1*04:02:01:02	DQA1*03:01:01	DQA1*05:05:01:02
**JG331**	DPA1*02:02:02:01	X	DPB1*05:01:01:01	DPB1*01:01:01:04	DQA1*03:03:01:02	DQA1*06:01:01:02
**JG332**	DPA1*01:03:01:01	DPA1*1:03:01:05	DPB1*02:01:02:05/416:01	DPB1*04:02:01:02/105:01/665:01	DQA1*03:01:01	DQA1*01:01:01:05
**JG127**	DPA1*01:03:01:04	DPA1*01:03:01:02	DPB1*04:01:01:06	DPB1*04:01:01:05	DQA1*01:01:01:05	DQA1*03:01:01
**JG128**	DPA1*02:06	DPA1*01:03:01:04	DPB1*04:01:01:06	DPB1*04:01:01:05	DQA1*01:02:01:03	DQA1*03:01:01
**JG129**	DPA1*02:01:08:02	DPA1*02:01:01:01	DPB1*01:01:01:01	DPB1*11:01:01:01	DQA1*03:03:01:01	DQA1*01:02:01:04
**JG143**	DPA1*02:01:08:02	DPA1*01:03:01:01	DPB1*01:01:01:01	DPB1*02:01:02:05	DQA1*04:01:01:04	DQA1*04:01:01:02
**JG147**	DPA1*02:02:02:01	DPA1*02:02:02:01	DPB1*05:01:01:01	DPB1*03:01:01:01	DQA1*03:03:01:03	DQA1*03:03:01:03
**JG149**	DPA1*01:03:01:04	DPA1*01:03:01:02	DPB1*04:01:01:06	DPB1*04:01:01:05	DQA1*05:01:01:02	DQA1*03:03:01:01
**JG159**	DPA1*01:03:01:02	DPA1*01:03:01:01	DPB1*16:01:01:01	DPB1*04:01:01:05	DQA1*03:03:01:01	DQA1*02:01:01:01
**JG262**	DPA1*01:03:01:03	DPA1*01:03:01:02	DPB1*03:01:01:01	DPB1*04:01:01:05	DQA1*03:01:01	DQA1*05:01:01:02
**JG272**	DPA1*01:03:01:02	DPA1*02:01:08:02	DPB1*104:01:01:01	DPB1*01:01:01:01	DQA1*01:02:01:03	DQA1*05:05:01:01
**Sample ID**	**DQB1/1**	**DQB1/2**	**DRB1/1**	**DRB1/2**	**DRB345/1**	**DRB345/2**
**JG330**	DQB1*03:02:01:01	DQB1*03:01:01:03	DRB1*04:01:01:03	DRB1*11:04:01	DRB4*01:03:01:10	DRB3*02:02:01:04
**JG331**	DQB1*04:02:01:09	DQB1*03:01:01:12	DRB1*04:05:01:01	DRB1*12:02:01:01	DRB4*01:03:01:01	DRB3*03:01:03
**JG332**	DQB1*03:02:01:01	DQB1*05:01:01:03	DRB1*04:01:01:03	DRB1*01:01:01	DRB4*01:03:01:10	Absent

**JG127**	DQB1*05:01:01:03	DQB1*03:02:01:02	DRB1*01:01:01	DRB1*04:04:01	DRB4*01:03:03	Absent
**JG128**	DQB1*06:02:01:01	DQB1*03:02:01:02	DRB1*15:01:01:05	DRB1*04:04:01	DRB5*01:01:01:01	DRB4*01:03:01:01
**JG129**	DQB1*02:02:01:02	DQB1*05:01:01:03	DRB1*09:01:02:01	DRB1*13:02:01:02	DRB4*01:01:01:01	DRB3*03:01:01:01
**JG143**	DQB1*04:02:01:08	DQB1*04:02:01:04	DRB1*08:11	DRB1*03:02:01	DRB3*01:62	Absent
**JG147**	DQB1*04:01:01:02	DQB1*04:01:01:02	DRB1*04:05:01:03	DRB1*04:05:01:02	DRB4*01:03:01:01	DRB4*01:03:01:01
**JG149**	DQB1*02:01:01	DQB1*03:01:01:01	DRB1*03:01:01:01	DRB1*04:01:01:01	DRB3*01:01:02:01	DRB4*01:03:01:10
**JG159**	DQB1*03:01:01:01	DQB1*02:02:01:01	DRB1*04:01:01:03	DRB1*07:01:01:04	DRB4*01:03:01:10	DRB4*01:01:01:01
**JG262**	DQB1*03:02:01:02	DQB1*02:01:01	DRB1*04:04:01	DRB1*03:01:01:01	DRB4*01:03:01:10	DRB3*01:01:02:01
**JG272**	DQB1*06:02:01:01	DQB1*03:01:01:03	DRB1*15:03:01:02	DRB1*13:03:01	DRB5*01:01:01:01	DRB3*01:01:02:03

Supplementary Table 4. Antibodies used in flow cytometry experiments.

Marker	Clone	Fluorescence	Company	Dilution
**CD3**	SK7	APC-H7	BD Biosciences	1:200
**CD4**	RPA-T4	FITC<br>Pacific Blue	BD Biosciences<br>BioLegend	1:200<br>1:200
**CD8**	RPA-T8	Alexa Fluor 700	BD Biosciences	1:200
**CD69**	FN50	PerCP-Cy5.5	BD Biosciences	1:200
**GPR56**	CG4	PE	BioLegend	1:200
**TCRγδ**	B1	FITC	BD Biosciences	1:200
**Granzyme K**	G3H69	Alexa Fluor 647	BD Biosciences	1:150
**Granzyme B**	GB11	PE<br>FITC	BD Biosciences	1:200<br>1:200
**CCR7**	150503	PE	BD Biosciences	1:150
**CD45RA**	HI100	Brilliant Violet 421<br>FITC	BioLegend<br>BD Biosciences	1:200<br>1:200
**PD-1**	NAT105	PerCP-Cy5.5	BioLegend	1:200
**TIM3**	F38-2E2	APC	BioLegend	1:200
**CD158b (KIR2DL2/L3)**	DX27	FITC	BioLegend	1:200
**His-tag**	J095G46	PE	BioLegend	1:100
**HLA-A,B,C**	W6/32	APC-Cy7	BioLegend	1:200
**Ki-67**	Ki-67	Brilliant Violet 421	BioLegend	1:100
**IFNγ**	4S.B3<br>B27<br>B27	APC-Cy7<br>PE<br>APC	BioLegend<br>BioLegend<br>BD Biosciences	1:200<br>1:200<br>1:150
**CD107a**	H4S3	APC-Cy7	BioLegend	1:200

Supplementary Table 5. CITE-seq antibodies used in 10X single cell RNA sequencing.

TotalSeqC-Barcode	Specificity	Clone	Reactivity	Barcode sequence
34	CD3	UCHT1	Human	CTCATTGTAACTCCT
45	CD4	SK3	Human	GAGGTTAGTGATGGA
46	CD8	SK1	Human	GCGCAACTTGATGAT
50	CD19	HIB19	Human	CTGGGCAATTACTCG
63	CD45RA	HI100	Human	TCAATCCTTCCGCTT
84	CD56	QA17A16	Human	TTCGCCGCATTGAGT
85	CD25	BC96	Human	TTTGTCCTGTACGCC
87	CD45RO	UCHL1	Human	CTCCGAATCATGTTG
88	PD-1	EH12.2H7	Human	ACAGCGCCGTATTTA
89	TIGIT	A15153G	Human	TTGCTTACCGCCAGA
139	TCRrd	B1	Human	CTTCCGATTCATTCA
140	CXCR3	G025H7	Human	GCGATGGTAGATTAT
143	CCR6	G034E3	Human	GATCCCTTTGTCACT
144	CXCR5	J252D4	Human	AATTCAACCGTCGCC
146	CD69	FN50	Human	GTCTCTTGGCTTAAA
147	CD62L	DREG-56	Human	GTCCCTGCAACTTGA
148	CD197/CCR7	G043H7	Human	AGTTCAGTCAACCGA
149	CD161	HP-3G10	Human	GTACGCAGTCCTTCT
154	CD27	O323	Human	GCACTCCTGCATGTA
159	HLA-DR	L243	Human	AATAGCGAGCAAGTA
165	NKG2D	1D11	Human	CGTGTTTGTTCCTCA
168	CD57	QA17A04	Human	AACTCCCTATGGAGG
179	CX3CR1	K0124E1	Human	AGTATCGTCTCTGGG
224	TCRab	IP26	Human	CGTAACGTAGAGCGA
246	CD122	TU27	Human	TCATTTCCTCCGATT
250	KLRG1	2F1/KLRG1	Human/Mouse	GTAGTAGGCTAGACC
386	CD28	CD28.2	Human	TGAGAACGACCCTAA
389	CD38	HIT2	Human	TGTACCCGCTTGTGA
390	IL-7Ra	A019D5	Human	GTGTGTTGTCCTATG
592	CD158b/KIR2DL2/L3	DX27	Human	GACCCGTAGTTTGAT
599	CD158e1/KIR3DL1	DX9	Human	GGACGCTTTCCTTGA

Supplementary Table 6. Gene list used to measure a score of synovial trafficking, cytotoxicity, proliferation or inflammation.

RA trafficking	Cytotoxicity	Proliferation	Inflammation
CXCR1	*GNLY*	*HMGB2*	*AGER*
CXCR2	*GZMA*	*CDK1*	*ALOX12*
CXCR3	*GZMB*	*NUSAP1*	*ALOX15*
CXCR4	*GZMH*	*UBE2C*	*ALOX5*
CXCR5	*GZMM*	*BIRC5*	*AREG*
CCR1	*GZMK*	*TPX2*	*ATF2*
CCR2	*NKG7*	*TOP2A*	*BCL2L1*
CCR3	*KLRB1*	*NDC80*	*BCL6*
CCR4	*KLRD1*	*CKS2*	*BIRC2*
CCR5	*KLRK1*	*NUF2*	*C1QA*
CCR6	*NCR1*	*CKS1B*	*C1QB*
CCR10	*SLAMF6*	*MKI67*	*C1R*
CX3CR1	*SLAMF7*	*TMPO*	*C1S*
CCL2	*TYROBP*	*CENPF*	*C2*
CCL3	*KIR3DL1*	*TACC3*	*C3*
CCL4	*KIR3DL2*	*SMC4*	*C3AR1*
CCL5		*CCNB2*	*C5*
CCL19		*CKAP2L*	*CCL17*
CCL20		*CKAP2*	*CCL19*
CXCL10		*AURKB*	*CCL2*
CXCL16		*BUB1*	*CCL20*
		KIF11	*CCL22*
		ANP32E	*CCL23*
		TUBB4B	*CCL24*
		GTSE1	*CCL3*
		KIF20B	*CCL4*
		HJURP	*CCL5*
		CDCA3	*CCR1*
		CDC20	*CCR2*
		TTK	*CCR3*
		CDC25C	*CCR4*
		KIF2C	*CCR7*
		RANGAP1	*CD4*

		NCAPD2	*CD40*
		DLGAP5	*CD40LG*
		CDCA2	*CD55*
		CDCA8	*CD86*
		ECT2	*CDC42*
		KIF23	*CEBPB*
		HMMR	*CFB*
		AURKA	*CFD*
		PSRC1	*CFL1*
		ANLN	*CREB1*
		LBR	*CSF1*
		CKAP5	*CSF2*
		CENPE	*CXCL10*
		CTCF	*CXCL3*
		NEK2	*CXCL9*
		G2E3	*CXCR1*
		GAS2L3	*CXCR2*
		CBX5	*CXCR4*
		CENPA	*CYSLTR1*
			CYSLTR2
			DAXX
			DDIT3
			ELK1
			FASLG
			FLT1
			FOS
			FXYD2
			GNAQ
			GNAS
			GNB1
			GRB2
			HDAC4
			HIF1A
			HLA-DRA
			HLA-DRB1
			HMGB1
			HMGB2
			HMGN1
			HRAS

			HSH2D
			HSPB1
			IFI44
			IFIT1
			IFIT2
			IFIT3
			IFNG
			IL10
			IL10RB
			IL12A
			IL13
			IL15
			IL17A
			IL18
			IL18RAP
			IL1A
			IL1B
			IL1R1
			IL1RAP
			IL1RN
			IL2
			IL21
			IL22
			IL22RA2
			IL23A
			IL23R
			IL3
			IL4
			IL5
			IL6
			IL6R
			IL7
			IRF1
			IRF3
			IRF5
			IRF7
			ITGB2
			JUN
			KEAP1

			LIMK1
			LTA
			LTB
			LTB4R
			LTB4R2
			LY96
			MAFF
			MAFG
			MAFK
			MAP2K1
			MAP2K4
			MAP2K6
			MAP3K1
			MAP3K5
			MAP3K7
			MAP3K9
			MAPK1
			MAPK14
			MAPK3
			MAPK8
			MAPKAPK2
			MAPKAPK5
			MASP2
			MAX
			MEF2A
			MEF2C
			MEF2D
			MKNK1
			MMP9
			MRC1
			MX1
			MX2
			MYC
			MYD88
			NFATC3
			NFE2L2
			NFKB1
			NLRP3
			NOD1

			NOD2
			NR3C1
			OAS2
			OASL
			PDGFA
			PLA2G4A
			PLCB1
			PPP1R12B
			PRKCA
			PRKCB
			PTGDR2
			PTGER2
			PTGER4
			PTGIR
			PTGS1
			PTGS2
			PTK2
			RAC1
			RAF1
			RAPGEF2
			RELA
			RELB
			RHOA
			RIPK1
			RIPK2
			ROCK2
			RPS6KA5
			SHC1
			SMAD7
			STAT1
			STAT2
			STAT3
			TBXA2R
			TCF4
			TGFB1
			TGFB3
			TGFBR1
			TLR1
			TLR2

			TLR3
			TLR4
			TLR5
			TLR6
			TLR8
			TLR9
			TNF
			TNFAIP3
			TNFSF14
			TOLLIP
			TRADD
			TRAF2
			TREM2
			TSLP
			TYROBP
