# Supplementary material for: Cytotoxic CD8+ T cells target citrullinated antigens in rheumatoid arthritis
Source: Nat Commun. 2023 Jan 19;14:319. doi: 10.1038/s41467-022-35264-8 (PMC9852471; doi:10.1038/s41467-022-35264-8)
Supplement: Supplementary file 3 — Reporting Summary [file 41467_2022_35264_MOESM3_ESM.pdf]

nature portfolio

Corresponding author(s): William H. Robinson
Last updated by author(s): 11-11-22

Reporting Summary

Nature Portfolio wishes to improve the reproducibility of the work that we publish. This form provides structure for consistency and transparency in reporting. For further information on Nature Portfolio policies, see our Editorial Policies and the Editorial Policy Checklist.

Statistics

For all statistical analyses, confirm that the following items are present in the figure legend, table legend, main text, or Methods section.

n/a	Confirmed	
☐	☒	The exact sample size (*n*) for each experimental group/condition, given as a discrete number and unit of measurement
☐	☒	A statement on whether measurements were taken from distinct samples or whether the same sample was measured repeatedly
☐	☒	The statistical test(s) used AND whether they are one- or two-sided<br>*Only common tests should be described solely by name; describe more complex techniques in the Methods section.*
☐	☒	A description of all covariates tested
☐	☒	A description of any assumptions or corrections, such as tests of normality and adjustment for multiple comparisons
☐	☒	A full description of the statistical parameters including central tendency (e.g. means) or other basic estimates (e.g. regression coefficient) AND variation (e.g. standard deviation) or associated estimates of uncertainty (e.g. confidence intervals)
☐	☒	For null hypothesis testing, the test statistic (e.g. *F*, *t*, *r*) with confidence intervals, effect sizes, degrees of freedom and *P* value noted<br>*Give P values as exact values whenever suitable.*
☒	☐	For Bayesian analysis, information on the choice of priors and Markov chain Monte Carlo settings
☒	☐	For hierarchical and complex designs, identification of the appropriate level for tests and full reporting of outcomes
☒	☐	Estimates of effect sizes (e.g. Cohen's *d*, Pearson's *r*), indicating how they were calculated

Our web collection on statistics for biologists contains articles on many of the points above.

Software and code

Policy information about availability of computer code

Data collection	Flow cytometry experiments were performed using a BD LSRFortessa or LSR II and Flow cytometric data was collected by BD FACSDiva (BD Biosciences). Single cell RNA sequencing was performed on Illumina Novaseq 6000.
Data analysis	Flow cytometric data was analyzed with Flowjo v.10.7.1 ; Using the cellranger mkfastq function (10X Genomics), base call files of for 5' gene expression libraries and V(D)J libraries were demultiplexed and converted into FASTQ files and mapped to the GRCh38 reference genome. TRA or TRB were annotated using the cellranger vdj function. R package Seurat (v.4.0.0) was used for normalization, scaling, integration, clustering, dimensionality reduction, differential expression analysis or visualization. Cell annotation was performed using the R package SingleR (v.1.1.7) to determine cellular identity. R package Harmony was used to normalize across batches. Analysis of gene ontology (GO) was performed using the clusterProfiler R package (v.3.10.1). scRepertoire R package v.1.145 was used to identify and analyze TCR clonotypes based on TCR alpha and beta chains and CDR3 sequences. The chord diagram to visualize the shared clones between each cluster was generated by getCirclize function using the R package circlize, and the heatmap was displayed with the R package ComplexHeatmap. Canonical correlation analysis (CCA) was used to compute the shared feature correlation structure that is conserved between the data sets, then mutual nearest neighbors (MNN) analysis used to identify transcriptionally matched cells in the datasets as described in FindTransferAnchors and TransferData functions in R package Seurat. GraphPad Prism v9 was used for the statistical analyses and plotting.<br>To identify differentially expressed genes (DEGs) in GZMB+ or GZMK+ clusters between ACPA+ RA and HC, we performed differential expression testing using the pseudobulk RNA expression.<br>We computed the pseudobulk gene expression for each cluster of each sample using the scater R package. Samples with less than 10 cells present in a cluster were excluded from the pseudobulk expression calculation of that cluster. Thereafter, the pseudobulk expression counts were normalized using DESeq2 R package. The normalized pseudobulk expression counts were used for differential gene expression analysis of ACPA+ RA vs. HC using DESeq2 R package. The calculated log2 fold change of gene expression in ACPA+ RA vs. HC was multiplied by the -log (FDR, false discovery rate) and used to rank the genes descending and perform gene-set enrichment analysis (GSEA) for GZMB+ cluster and

GZMK+ cluster. The normalized enrichment score (NES) and adjusted P value for multiple testing were used to define the enriched biological process pathways in either GZMB+ or GZMK+ clusters. The gene sets of gene ontology were obtained from Molecular Signatures Database (v7.5.1) and the GSEA was performed using the fgsea R package.
Here is the version of each R package we used.
ComplexHeatmap_2.12.1, circlize_0.4.15, fgsea_1.22.0, DESeq2_1.36.0, scater_1.24.0, ggplot2_3.3.6, scuttle_1.6.3
SingleCellExperiment_1.18.0, SummarizedExperiment_1.26.1, Biobase_2.56.0, GenomicRanges_1.48.0, GenomeInfoDb_1.32.4
IRanges_2.30.1, S4Vectors_0.34.0, BiocGenerics_0.42.0, MatrixGenerics_1.8.1, matrixStats_0.62.0, sctransform_0.3.4, harmony_0.1.0
Rcpp_1.0.9, scds_1.12.0

For manuscripts utilizing custom algorithms or software that are central to the research but not yet described in published literature, software must be made available to editors and reviewers. We strongly encourage code deposition in a community repository (e.g. GitHub). See the Nature Portfolio guidelines for submitting code & software for further information.

Data

Policy information about availability of data

All manuscripts must include a data availability statement. This statement should provide the following information, where applicable:

- Accession codes, unique identifiers, or web links for publicly available datasets
- A description of any restrictions on data availability
- For clinical datasets or third party data, please ensure that the statement adheres to our policy

The raw and processed single cell RNA and V(D)J data generated in this study have been deposited in the Sequence Read Archive (SRA) database under accession code PRJNA900189 [https://dataview.ncbi.nlm.nih.gov/object/PRJNA900189?reviewer=u4f41jqemv4sojlqpv6psoslda]. The differentially expressed gene data, CITE-seq list, or gene list used to measure scores of cytotoxic, proliferation or RA trafficking generated in this study are provided in the Supplementary Information.

Human research participants

Policy information about studies involving human research participants and Sex and Gender in Research.

Reporting on sex and gender: In this study, sex and gender were not determined as a factor even though most of RA samples were collected from male because we recruited RA patients at VA Palo Alto. We didn't see any difference when we analyzed based on sex difference.

Population characteristics: 59 RA patients and 30 healthy individuals were included in this study. 45 with ACPA positive and 14 with ACPA negative patients. ACPA positivity was tested by serum ELISA. Reference range for ACPA level; Less than 20 is ACPA Negative. 55 male and 4 female were included. Patient details are listed in Supplementary Table 1.

Recruitment: Among RA patients who came to VA Palo Alto Health Care System or Stanford Hospital, those who voluntarily participated in this study were recruited. RA blood samples were collected under institutional review board (IRB) approved protocols and after written informed consent at VA Palo Alto Health Care System and Stanford University. Healthy samples were obtained through the Stanford Blood Center. Paired RA blood and synovium samples were collected at HSS New York. There are no self- selection bias or other biases that may be present.

Ethics oversight: All experimental protocols were approved by the institutional review board of Stanford University and VA Palo Alto (IRB 3780).

Note that full information on the approval of the study protocol must also be provided in the manuscript.

Field-specific reporting

Please select the one below that is the best fit for your research. If you are not sure, read the appropriate sections before making your selection.

[x] Life sciences [ ] Behavioural & social sciences [ ] Ecological, evolutionary & environmental sciences

For a reference copy of the document with all sections, see nature.com/documents/nr-reporting-summary-flat.pdf

Life sciences study design

All studies must disclose on these points even when the disclosure is negative.

Sample size: Single-cell RNA sequencing includes n=18 individuals (ACPA+ RA=12, Healthy control=6). 16,000 CD8+ T cells were recovered by normalization step from all individuals. 10,400 paired TCRab sequences were recovered. The exact sample size of each experiment is described in the relevant Figure legends. Sample sizes were determined based on previous papers, experimental trials or meaningful statistics.
For paired samples, we included 3 ACPA+ RA blood and 4 synovium and 9,360 TCR ab sequences were recovered.
Predetermination of sample size calculation was not performed as we included as many sizes as for statistical analysis.

Data exclusions: Data with >10% of mitochondrial genes or low (<200) gene counts for single cell RNA seq were excluded from the cell count. Samples with low cell viability were excluded from flow cytometric analysis.

Replication: All experiments were performed with at least two biological replicates. For single cell RNA seq, multiple batches from different individuals

	were merged using a MNN based batch correction with R package Harmony. We included quantification results if we provide a representative image. All results presented in the manuscript indicate a high data reproducibility.
Randomization	Randomization was not relevant to this study as this study does not explore group differences. Healthy controls were selected by random participants (no RA symptoms).
Blinding	Flow cytometric analysis was blinded by assigning unique number of samples. The investigators were not blinded to single cell RNA seq data as ACPA+ RA samples were chosen by ACPA level in serum and a clinical outcome association was performed. Investigators were blinded to group allocation during experiments and analysis of the results.

Reporting for specific materials, systems and methods

We require information from authors about some types of materials, experimental systems and methods used in many studies. Here, indicate whether each material, system or method listed is relevant to your study. If you are not sure if a list item applies to your research, read the appropriate section before selecting a response.

Materials & experimental systems

n/a	Involved in the study
☐	☒ Antibodies
☐	☒ Eukaryotic cell lines
☒	☐ Palaeontology and archaeology
☒	☐ Animals and other organisms
☐	☒ Clinical data
☒	☐ Dual use research of concern

Methods

n/a	Involved in the study
☒	☐ ChIP-seq
☐	☒ Flow cytometry
☒	☐ MRI-based neuroimaging

Antibodies

Antibodies used

For flow cytometry; (Dilution 1:200, otherwise noted)
CD3 (clone: SK7, APC-H7, BD Biosciences #560176)
CD4 (clone: RPA-T4, FITC, BD Biosciences #560607)
CD4 (clone: RPA-T4, Pacific Blue, BioLegend #300524)
CD8 (clone: RPA-T8, Alexa Fluor 700, BD Biosciences #557945)
CD69 (clone: FN50, PerCP-Cy5.5, BD Biosciences #560738)
GPR56 (clone: CG4, PE, BioLegend #358203)
TCRγδ (clone: B1, FITC, BD Biosciences #561995)
Granzyme K (clone: G3H69, Alexa Fluor 647, BD Biosciences #566655, 1:150)
Granzyme B (clone: GB11, PE, BD Biosciences #561142)
Granzyme B (clone: GB11, FITC, BD Biosciences #561998)
CCR7 (clone: 150503, PE, BD Biosciences #560765, 1:150)
CD45RA (clone: HI100, Brilliant Violet 421, BioLegend #304129)
CD45RA (clone: HI100, FITC, BD Biosciences #555488)
PD-1 (clone: NAT105, PerCP-Cy5.5, BioLegend #367409)
TIM3 (clone: F38-2E2, APC, BioLegend #345011)
CD158b (KIR2DL2/L3) (clone: DX27, FITC, BioLegend #312603)
His-tag (clone: J095G46, PE, BioLegend #362603, 1:100)
HLA-A,B,C (clone: W6/32, APC-Cy7, BioLegend #311425)
Ki-67 (clone: Ki-67, Brilliant Violet 421, BioLegend #350506, 1:100)
IFNγ (clone: 4S.B3, APC-Cy7, BioLegend #502529)
IFNγ (clone: B27, PE, BioLegend #506507)
IFNγ (clone: B27, APC, BD Biosciences #562017, 1:150)
CD107a (clone: H4S3, APC-Cy7, BioLegend #328630)

For cell culture;
Purified NA/LE Mouse Anti-Human CD3 (clone: OKT3, BD Biosciences #566685, 1 ug/ml)
Purified NA/LE Mouse Anti-Human CD28 (clone: CD28.2, BD Biosciences #555725, 1 ug/ml)
Purified anti-human CD49d Antibody (clone: 9F10, BioLegend #304301, 1 ug/ml)
Ultra-LEAF™ Purified anti-human HLA-A,B,C Antibody (clone: W6/32, BioLegend #311428, 1 ug/ml)
Purified anti-human CD8 Antibody (clone: SK1, BioLegend #344702, 1 ug/ml)

For CITE-seq; (Dilution 1:100)
TotalSeqC-Barcode Specificity Clone Reactivity Barcode sequence
34 CD3 UCHT1 Human CTCATTGTAACTCCT
45 CD4 SK3 Human GAGGTTAGTGATGGA
46 CD8 SK1 Human GCGCAACTTGATGAT
50 CD19 HIB19 Human CTGGGCAATTACTCG

63 CD45RA HI100 Human TCAATCCTTCCGCTT
84 CD56 QA17A16 Human TTCGCCGCATTGAGT
85 CD25 BC96 Human TTTGTCCTGTACGCC
87 CD45RO UCHL1 Human CTCCGAATCATGTTG
88 PD-1 EH12.2H7 Human ACAGCGCCGTATTTA
89 TIGIT A15153G Human TTGCTTACCGCCAGA
139 TCRrd B1 Human CTTCCGATTCATTCA
140 CXCR3 G025H7 Human GCGATGGTAGATTAT
143 CCR6 G034E3 Human GATCCCTTTGTCACT
144 CXCR5 J252D4 Human AATTCAACCGTCGCC
146 CD69 FN50 Human GTCTCTTGGCTTAAA
147 CD62L DREG-56 Human GTCCCTGCAACTTGA
148 CD197/CCR7 G043H7 Human AGTTCAGTCAACCGA
149 CD161 HP-3G10 Human GTACGCAGTCCTTCT
154 CD27 O323 Human GCACTCCTGCATGTA
159 HLA-DR L243 Human AATAGCGAGCAAGTA
165 NKG2D 1D11 Human CGTGTTTGTTCCTCA
168 CD57 QA17A04 Human AACTCCCTATGGAGG
179 CX3CR1 K0124E1 Human AGTATCGTCTCTGGG
224 TCRab IP26 Human CGTAACGTAGAGCGA
246 CD122 TU27 Human TCATTTCCTCCGATT
250 KLRG1 2F1/KLRG1 Human/Mouse GTAGTAGGCTAGACC
386 CD28 CD28.2 Human TGAGAACGACCCTAA
389 CD38 HIT2 Human TGTACCCGCTTGTGA
390 IL-7Ra A019D5 Human GTGTGTTGTCCTATG
592 CD158b/KIR2DL2/L3 DX27 Human GACCCGTAGTTTGAT
599 CD158e1/KIR3DL1 DX9 Human GGACGCTTTCCTTGA

Validation

Antibodies used in this study are commercially available and validated by their respective manufacturers.
For flow cytometry;
CD3 (APC-H7, BD Biosciences #560176) - https://www.bdbiosciences.com/en-us/products/reagents/flow-cytometry-reagents/research-reagents/single-color-antibodies-ruo/apc-h7-mouse-anti-human-cd3.560176
CD4 (FITC, BD Biosciences #561005) - https://www.bdbiosciences.com/en-us/products/reagents/flow-cytometry-reagents/research-reagents/single-color-antibodies-ruo/fitc-mouse-anti-human-cd4.561005
CD4 (Pacific Blue, BioLegend #300524) - https://www.biolegend.com/en-us/search-results/pacific-blue-anti-human-cd4-antibody-2850?GroupID=BLG7755
CD8 (Alexa Fluor 700, BD Biosciences #557945) - https://www.bdbiosciences.com/en-us/products/reagents/flow-cytometry-reagents/research-reagents/single-color-antibodies-ruo/alexa-fluor-700-mouse-anti-human-cd8.561026
CD69 (PerCP-Cy5.5, BD Biosciences #560738) - https://www.bdbiosciences.com/en-us/products/reagents/flow-cytometry-reagents/research-reagents/single-color-antibodies-ruo/percp-cy-5-5-mouse-anti-human-cd69.560738
GPR56 (PE, BioLegend #358203) - https://www.biolegend.com/en-us/products/pe-anti-human-gpr56-antibody-8542?GroupID=BLG11558
TCRγδ (FITC, BD Biosciences #561995) - https://www.bdbiosciences.com/en-us/products/reagents/flow-cytometry-reagents/research-reagents/single-color-antibodies-ruo/fitc-mouse-anti-human-tcr.561995
Granzyme K (Alexa Fluor 647, BD Biosciences #566655) - https://www.bdbiosciences.com/en-us/products/reagents/flow-cytometry-reagents/research-reagents/single-color-antibodies-ruo/alexa-fluor-647-mouse-anti-human-granzyme-k.566655
Granzyme B (PE, BD Biosciences #561142) - https://www.bdbiosciences.com/en-us/products/reagents/flow-cytometry-reagents/research-reagents/single-color-antibodies-ruo/pe-mouse-anti-human-granzyme-b.561142
Granzyme B (FITC, BD Biosciences #561998) - https://www.bdbiosciences.com/en-us/products/reagents/flow-cytometry-reagents/research-reagents/single-color-antibodies-ruo/fitc-mouse-anti-human-granzyme-b.560211
CCR7 (PE, BD Biosciences #560765) - https://www.bdbiosciences.com/en-us/products/reagents/flow-cytometry-reagents/research-reagents/single-color-antibodies-ruo/pe-mouse-anti-human-cd197-ccr7.560765
CD45RA (Brilliant Violet 421, BioLegend #304129) - https://www.biolegend.com/en-us/products/brilliant-violet-421-anti-human-cd45ra-antibody-7200?GroupID=GROUP658
CD45RA (FITC, BD Biosciences #555488) - https://www.bdbiosciences.com/en-us/products/reagents/flow-cytometry-reagents/research-reagents/single-color-antibodies-ruo/fitc-mouse-anti-human-cd45ra.561882
PD-1 (PerCP-Cy5.5, BioLegend #367409) - https://www.biolegend.com/en-us/search-results/percp-cyanine5-5-anti-human-cd279-pd-1-antibody-12736?GroupID=BLG5466
TIM3 (APC, BioLegend #345011) - https://www.biolegend.com/en-us/products/apc-anti-human-cd366-tim-3-antibody-8302
CD158b (KIR2DL2/L3) (FITC, BioLegend #312603) - https://www.biolegend.com/en-us/products/fitc-anti-human-cd158b-kir2dl2-l3-nkat2-antibody-2280?GroupID=BLG8440
His-tag (PE, BioLegend #362603) - https://www.biolegend.com/en-us/products/pe-anti-his-tag-antibody-9861
HLA-A,B,C (APC-Cy7, BioLegend #311425) - https://www.biolegend.com/en-us/products/apc-cyanine7-anti-human-hla-a-b-c-antibody-7058?GroupID=BLG5954
Ki-67 (Brilliant Violet 421, BioLegend #350506) - https://www.biolegend.com/en-us/products/brilliant-violet-421-anti-human-ki-67-antibody-7146?GroupID=GROUP28
IFNγ (APC-Cy7, BioLegend #502529) - https://www.biolegend.com/en-us/products/apc-cyanine7-anti-human-ifn-gamma-antibody-6965
IFNγ (PE, BioLegend #506507) - https://www.biolegend.com/en-us/products/pe-anti-human-ifn-gamma-antibody-1536

IFNγ (APC, BD Biosciences #562017) - https://www.bdbiosciences.com/en-us/products/reagents/flow-cytometry-reagents/research-reagents/single-color-antibodies-ruo/apc-mouse-anti-human-ifn.562017
CD107a (APC-Cy7, BioLegend #328630) - https://www.biolegend.com/en-us/products/apc-cyanine7-anti-human-cd107a-lamp-1-antibody-8557?GroupID=BLG10252

For cell culture;
Purified NA/LE Mouse Anti-Human CD3 (BD Biosciences #566685) - https://www.bdbiosciences.com/en-us/products/reagents/flow-cytometry-reagents/research-reagents/single-color-antibodies-ruo/purifed-na-le-mouse-anti-human-cd3.566685
Purified NA/LE Mouse Anti-Human CD28 (BD Biosciences #555725) - https://www.bdbiosciences.com/en-us/products/reagents/flow-cytometry-reagents/research-reagents/single-color-antibodies-ruo/purified-na-le-mouse-anti-human-cd28.555725
Purified anti-human CD49d Antibody (BioLegend #304301) - https://www.biolegend.com/en-us/products/purified-anti-human-cd49d-antibody-586?GroupID=BLG2458
Ultra-LEAF™ Purified anti-human HLA-A,B,C Antibody (BioLegend #311428) - https://www.biolegend.com/en-us/products/ultra-leaf-purified-anti-human-hla-a-b-c-antibody-8095
Purified anti-human CD8 Antibody (BioLegend #344702) - https://www.biolegend.com/en-us/products/purified-anti-human-cd8-antibody-6144

For CITE-seq; followed recommended protocol from BioLegend.

Eukaryotic cell lines

Policy information about cell lines and Sex and Gender in Research

Cell line source(s)	DLD-1 (ATCC, ATCC® CCL-221)
Authentication	Cell from commercial source was distributed with certificates of authentication using STR profiling.
Mycoplasma contamination	Regular mycoplasma testing was done for DLD-1. It tested negative for mycoplasma contamination.
Commonly misidentified lines (See ICLAC register)	No commonly misidentified cell line

Clinical data

Policy information about clinical studies

All manuscripts should comply with the ICMJEguidelines for publication of clinical research and a completedCONSORT checklist must be included with all submissions.

Clinical trial registration	n/a
Study protocol	n/a
Data collection	n/a
Outcomes	n/a

Flow Cytometry

Plots

Confirm that:

- [x] The axis labels state the marker and fluorochrome used (e.g. CD4-FITC).
- [x] The axis scales are clearly visible. Include numbers along axes only for bottom left plot of group (a 'group' is an analysis of identical markers).
- [x] All plots are contour plots with outliers or pseudocolor plots.
- [x] A numerical value for number of cells or percentage (with statistics) is provided.

Methodology

Sample preparation	For cellular profiling of CD8+ T cells, whole blood was collected and PBMCs isolated using Ficoll-Paque density gradient centrifugation (Sigma Aldrich). Cells were cryopreserved in Recovery Cell Culture Freezing Medium (Thermo Fisher Scientific). Thawed PBMCs were stabilized at 37 °C overnight prior to staining with Fixable Viability Stain 510 (BD Bioscience), followed by fluorophore-conjugated antibodies targeting surface molecules in Stain Buffer (BD Bioscience) (Supplementary Table 4). For intracellular staining, PBMCs were re-stimulated with Cell Stimulation Cocktail (Thermo Fisher Scientific), fixed and permeabilized, then stained with intracellular molecule-targeting antibodies.
Instrument	BD LSRFortessa/ LSR II flow cytometer (BD Biosciences)
Software	The stained cells were measured using a BD LSR Fortessa or LSR II flow cytometer and analyzed with FlowJo v.10.7.1 (Treestar).

Cell population abundance	The population of CD8+ T cells in PBMCs consisted of 2-40%. CD8+ or CD3+ T cells were isolated by magnetic based EasySEP isolation kit (StemCell Biotechnologies).
Gating strategy	Details of the gating strategy are represented in Extended Data Fig. 1. Briefly, for every flow cytometric data, FSC-A and SSC-A gates were used to identify lymphocytes. Using FSC-A and FSC-H gating, singlet cells were identified. Then, we applied live/dead stain (Fixable stain 510) to select for live cells. CD8+ T cells from PBMCs were gated on CD3+/CD4-. For gating of positive population, we gated a distinctly positive population compared to negative population.

☒ Tick this box to confirm that a figure exemplifying the gating strategy is provided in the Supplementary Information.
